# Monitoring and Risk Prediction of Low-Temperature Stress in Strawberries through Fusion of Multisource Phenotypic Spatial Variability Features

**DOI:** 10.1016/j.plaphe.2025.100041

**Published:** 2025-05-06

**Authors:** Nan Jiang, Zaiqiang Yang, Hanqi Zhang, Chengjing Zhang, Canyue Wang, Na Wang, Chao Xu

**Affiliations:** a School of Ecology and Applied Meteorology, Nanjing University of Information Science & Technology, Nanjing, 210044, PR China; b Institute of Horticulture, Jiangxi Academy of Agricultural Sciences, Nanchang, 330299, PR China

**Keywords:** Cold stress monitoring and early warning, Explainable machine learning, Fragaria × ananassa Duch., Phenotypic spatial variability features, Photosynthetic physiology

## Abstract

Capturing crop physiological information by phenotyping is a key trend in smart agriculture. However, current studies underutilize spatial structural information in phenotypic imaging. To evaluate the feasibility of crop cold stress monitoring based on phenotypic spatial variability, we conducted controlled experiments on ‘Toyonoka’ strawberry plants under four dynamic cooling gradients and three stress durations and analyzed the dependence of their photosynthetic physiology and phenotypic traits on temperature-time interactions. The results revealed that NPQ/1D-Parallel/TENT, Y(NO)/2D-Region/INEM, and qP/1D-Parallel/TENT presented the highest mutual information, with the maximum net photosynthetic rate (P_max_), relative electrolyte conductivity (REC), and total chlorophyll content (Chl_a ​+ ​b_), respectively. The difference between the Photosynthetic Physiological Potential Index (PPPI) and relative negative accumulated temperature (RNAT)/650 effectively was used to calculate the cold damage risk (CDRI). An XGBoost-based model integrating the PPPI and RNAT outperformed AdaBoost and RandomForest, achieving an R^2^ of 0.98, an RMSE of 0.337, a classification accuracy of 92.13 ​%, and a Kappa coefficient of 0.904. qP/1D-Parallel/TENT contributed the most to the model. This study provides a scientific basis for phenotypic information mining and agro-meteorological disaster monitoring.

## 1 Introduction

Strawberry (*Fragaria* ​× ​*ananassa* Duch.) is rich in anthocyanins, phenolics, and flavonoids, which contribute to antioxidative activity, immune enhancement, and aging delay, making it one of the most popular fruits worldwide [[1], [2], [3]]. Optimal growth occurs at 15–25 ​°C, whereas low temperatures (T ​≥ ​0 ​°C) suppress flower bud differentiation and gametophyte development, compromising fruit quality and yield [4,5]. As strawberries are cultivated primarily from late autumn to early spring, temperatures drop below 5 ​°C in more than 90 ​% of the regions where strawberries are grown during this period due to solar radiation, atmospheric circulation, and ocean currents [[6], [7], [8]]. Consequently, preventing cold damage remains a persistent challenge for the strawberry industry. Today, the increasing frequency and intensity of extreme weather events, including severe cold spells, have become hallmarks of global climate change. Avoiding or mitigating agricultural meteorological disasters caused by such events is an urgent and critical issue [9,10].

Photosynthesis is the fundamental energy source for plants, providing the essential basis for their survival, growth, and development [11,12]. The maximum net photosynthetic rate (P_max_), a direct indicator of a plant's photosynthetic performance, is widely used in studies of both biotic and abiotic stresses and serves as a measure of stress severity [[13], [14], [15]]. Chlorophyll plays a central role in the light-dependent reactions of photosynthesis, absorbing light energy and transferring it to the reaction centers of Photosystem II (PSII) and Photosystem I (PSI) for conversion into chemical energy [16]. The Chlorophyll content (Chl_a ​+ ​b_) is frequently employed as a key indicator in studies of physiological responses to environmental changes, as it reflects a plant's photosynthetic potential [[17], [18], [19]]. Under environmental stress, plant cell membranes may rupture, leading to the leakage of intracellular electrolytes such as K^+^ and Cl^−^, which exacerbates cellular damage [20,21]. Measuring leaf relative electrolyte conductivity (REC) provides insight into the integrity of photosynthetic tissues, offering a comprehensive assessment of stress severity.

Since the turn of the 21st century, advances in optical sensor technology and the widespread adoption of computing technologies have brought phenotypic information to the forefront of plant physiological research. Owing to their quantitative, macroscopic, and non-contact advantages, phenotypic data have become crucial sources of plant physiological information [22,23]. Hyperspectral imaging, a classic representative of plant phenotyping, excels at capturing changes in leaf structure, pigment content, and elemental composition through the reflection characteristics of different spectral bands. This technology is frequently employed in various applications, such as physiological parameter inversion, pest and disease diagnosis, and productivity estimation through spectral calculations [[24], [25], [26]]. Spectral data offer comprehensive and stable insights into plant physiological status [27]. Moreover, chlorophyll fluorescence imaging has emerged as a new focus in plant phenotyping research. It reveals the spatial distribution of PSII efficiency, photoprotection capacity, and photodamage within a plant based on the competitive dynamics among photochemical reactions, chlorophyll fluorescence excitation, and thermal dissipation [28,29]. Compared with spectral data, chlorophyll fluorescence reflects the immediate state of a plant's photosynthetic system and responds more sensitively and rapidly to physiological changes. This makes it particularly effective for the early detection of drought, disease, and other stressors [[30], [31], [32]]. Each data source clearly has distinct advantages [33].

While individual data sources provide limited physiological insights, fusing multiple sources can increase the richness and reliability of plant physiological information [34]. The fusion of RGB and spectral imaging has been shown to increase the detection efficiency for potential banana diseases and apple pests, whereas the integration of multispectral and hyperspectral imaging has improved nitrogen status assessment in tea plants [[35], [36], [37]]. In recent years, studies combining chlorophyll fluorescence and reflectance-based phenotyping have emerged, demonstrating the effectiveness of reflectance and fluorescence imaging in diagnosing salt stress in cotton, identifying heavy metal stress in rice, and evaluating postharvest pineapple quality [[38], [39], [40]]. Similarly, integrating solar-induced chlorophyll fluorescence (SIF) with reflectance improves the accuracy of rice photosynthetic capacity estimation beyond either data source alone [41]. Moreover, the fusion of spectral reflectance and texture information has shown superior performance in estimating chlorophyll content in rice and apples, highlighting the role of spatial structural traits as key external manifestations of plant physiological dynamics [42,43]. However, these studies predominantly average pixel parameters within the region of interest (ROI), such as leaf tissues. We argue that this approach merely amplifies traditional point-based spectral and fluorescence measurements, overlooking the most valuable aspect of phenotypic information—spatial variability. In contrast, Dong et al. reported heterogeneity in the distribution of chlorophyll fluorescence parameters along transverse sections of tomato leaves under cold stress but did not further explore the quantitative significance of this heterogeneity [44].

In summary, the quantification of plant phenotypic spatial variability across different dimensions and orientations, as well as its dynamic response to physiological stress, remains unexplored. Current phenotyping-based plant stress diagnosis studies are often anchored to subjective labels, with few quantitative links to actual physiological indicators [45,46]. Moreover, agricultural meteorological disaster early warning systems remain predominantly reliant on time series meteorological data, with no studies exploring the feasibility of a purely phenotypic approach for crop disaster risk prediction. To address these scientific questions and practical production needs, this study explores the feasibility of using spatial variability features derived from chlorophyll fluorescence and hyperspectral phenotyping across different dimensions and distribution orientations to indicate changes in strawberry photosynthetic potential under varying low-temperature gradients and stress durations. By fusing multisource phenotypic spatial variability features closely tied to plant photosynthetic physiology, we aimed to effectively monitor strawberry low-temperature stress and predict disaster risk.

## 2 Materials and methods

### 2.1 Overall technical approach

**Fig. 1:**
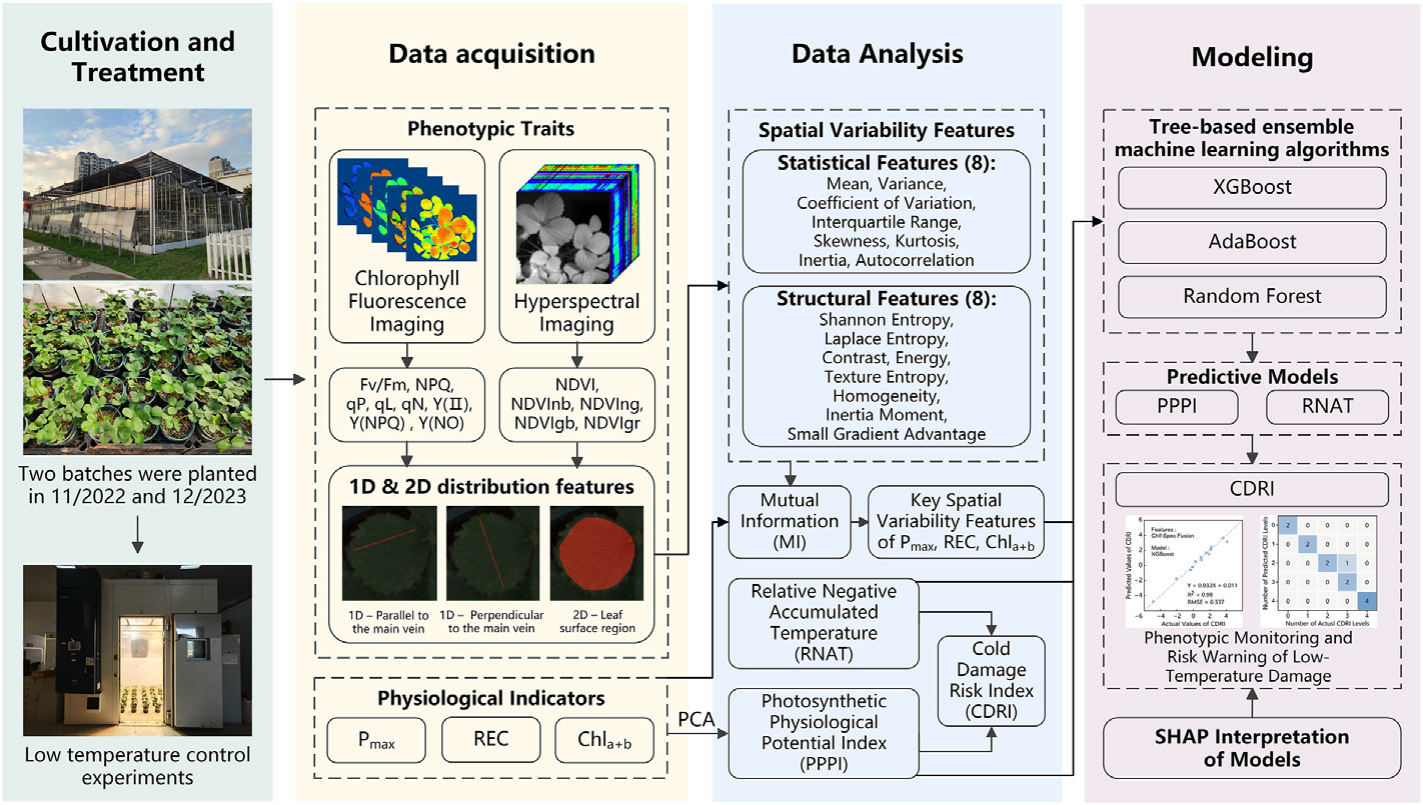
Overall technical approach diagram.

This study involved strawberry cultivation experiments and low-temperature control tests to collect two types of phenotypic data, chlorophyll fluorescence imaging and hyperspectral imaging, along with three physiological photosynthetic parameters—P_max_, REC, and Chl_a ​+ ​b_. We constructed the Photosynthetic Physiological Potential Index (PPPI) and used it in conjunction with the Relative Negative Accumulated Temperature (RNAT) to develop the Cold Damage Risk Index (CDRI). Key phenotypic spatial variability features highly dependent on photosynthetic physiological parameters were selected to establish inversion models for PPPI and RNAT. The CDRI prediction was then calculated based on these two models, enabling the development of a strawberry low-temperature stress monitoring and disaster risk prediction model through the fusion of multisource phenotypic spatial variability features. Finally, the model was analyzed using SHAP (SHapley Additive exPlanations) for interpretability. The overall technical approach is illustrated in Fig. 1.

### 2.2 Plant materials

The short-day strawberry variety ‘Toyonoka’ was selected as the experimental material for this study. The plants were purchased in two batches, in November 2022 and December 2023, from a strawberry cultivation base in Xuzhou, Jiangsu. These were vigorous seedlings of the current year that had not yet flowered. Healthy strawberry plants with 5–6 well-formed, dark green, and uniformly textured functional leaves, a robust main stem and petioles, and an approximate height of 15 ​cm were selected. These plants were subsequently transplanted into plastic horticultural pots with dimensions of 18 ​cm (upper diameter) ​× ​15.5 ​cm (lower diameter) ​× ​19 ​cm (height). Each pot contained one experimental strawberry plant and was filled with approximately 3.5 ​L of a substrate composed of a mixture of peat soil, rice husks, sand, perlite, and vermiculite at a 3:2:1:1:1 (v:v:v:v) ratio. No base fertilizer was applied at planting; instead, supplementary fertilization was administered after the plants had acclimated. A total of 78 ‘Toyonoka’ strawberry plants were used in this study, with each experimental group comprising 6 treated plants. Among these, 5 plants were used for physiological analysis and model development, while 1 plant served as an independent test sample, with control treatments and measurements identical to those of the other 5 plants.

### 2.3 Experimental management and treatment

Prior to initiating the controlled environmental experiments, all the strawberry plants were acclimated and cultivated in a Venlo-type glass greenhouse at Nanjing University of Information Science and Technology (NUIST) (32°12′N, 118°42′E). During this period, the plants were maintained under uniform microclimate and water-nutrient management conditions. The greenhouse's ventilation, misting, shading, and irrigation systems were fully automated, keeping the ambient temperature between 15 and 25 ​°C and the relative humidity at 60–80 ​%. During the 8-day acclimation period, no water or fertilizer was supplied. After acclimation, drip irrigation was applied every 3–4 days to maintain the soil moisture at 60–70 ​%, with a fertilizer application rate of 30 ​kg/ha (N:P:K ​= ​20:20:20), and irrigation was scheduled from 16:00–17:00. Three weeks after transplanting, the strawberry plants that had reached the flowering stage (with at least one open flower) and displayed uniform growth were selected for the temperature treatment experiments.

The experiment was conducted in two batches from November to December 2022 and from December 2023 to January 2024 ​at the NUIST Agro-Meteorological Experimental Station. Four low-temperature gradients were set up: 19/9 ​°C (daytime high/nighttime low), 16/6 ​°C, 13/3 ​°C, and 10/0 ​°C, with a control group maintained at 25/15 ​°C. Each treatment and control group was subjected to three durations: 3 days, 6 days, and 9 days. Detailed information for each experimental group is provided in Table 1.

**Table 1:** Experimental groups details.

Treatment Temperature (Daily Maximum Temperature/Daily Minimum Temperature)	Treatment Duration	Treatment Marker	Treatment Date (Start Date - End Date)
25 ​°C/15 ​°C (Control Temperature)	3d	CK	November 22, 2022∼November 25, 2022, December 24, 2023∼December 27, 2023^a^
	6d		November 22, 2022∼November 28, 2022, December 24, 2023∼December 30, 2023
	9d		November 22, 2022∼December 1, 2022, December 24, 2023∼January 2, 2024
19/9 ​°C	3d	T1D3	December 1, 2022∼December 4, 2022, December 24, 2023∼December 27, 2023
	6d	T1D6	December 1, 2022∼December 7, 2022, December 24, 2023∼December 30, 2023
	9d	T1D9	December 1, 2022∼December 10, 2022, December 24, 2023∼January 2, 2024
16/6 ​°C	3d	T2D3	November 22, 2022∼November 25, 2022, January 2, 2024∼January 5, 2024
	6d	T2D6	November 22, 2022∼November 28, 2022, January 2, 2024∼January 8, 2024
	9d	T2D9	November 22, 2022∼December 1, 2022, January 2, 2024∼January 11, 2024
13/3 ​°C	3d	T3D3	December 1, 2022∼December 4, 2022, December 24, 2023∼December 27, 2023
	6d	T3D6	December 1, 2022∼December 7, 2022, December 24, 2023∼December 30, 2023
	9d	T3D9	December 1, 2022∼December 10, 2022, December 24, 2023∼January 2, 2024
10/0 ​°C	3d	T4D3	November 22, 2022∼November 25, 2022, January 2, 2024∼January 5, 2024
	6d	T4D6	November 22, 2022∼November 28, 2022, January 2, 2024∼January 8, 2024
	9d	T4D9	November 22, 2022∼December 1, 2022, January 2, 2024∼January 11, 2024

a Each experimental group consisted of 3 replicates per batch, with a total of 6 replicates across the two batches.

**Fig. 2:**
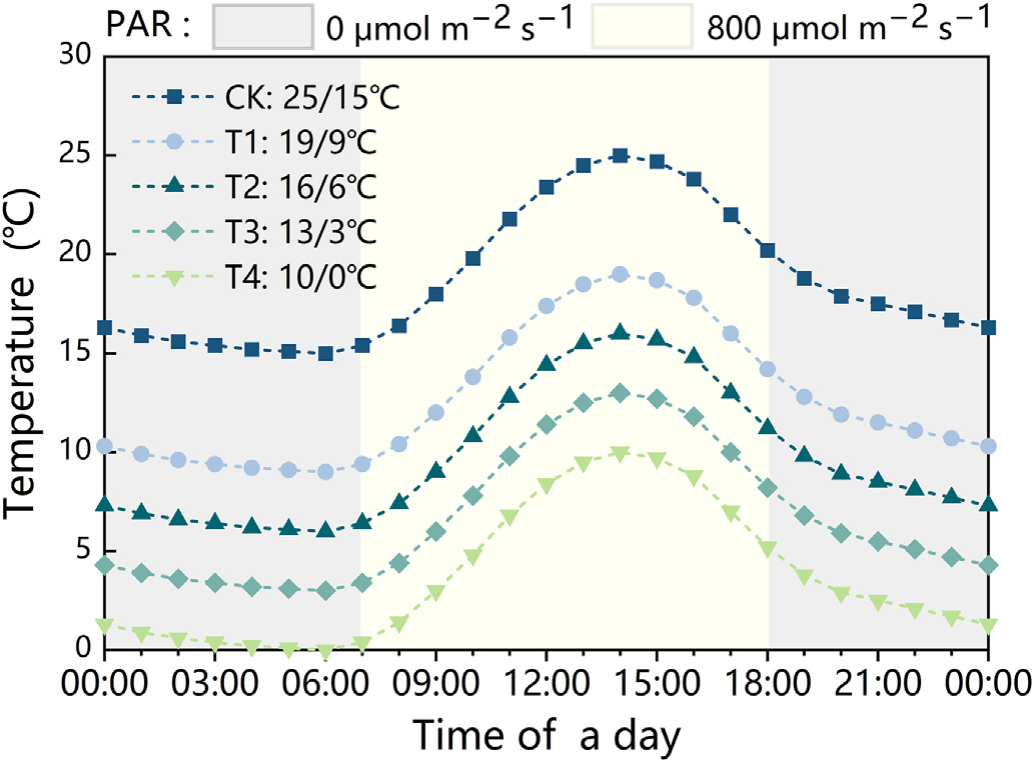
Diurnal temperature and light settings in the growth chamber.

The controlled environment experiments were conducted in a PGC-FLEX walk-in growth chamber (Conviron, Canada). The diurnal temperature and photoperiod settings of the chamber are illustrated in Fig. 2, with hourly temperature variations designed to simulate the typical daily temperature fluctuations observed in a solar greenhouse [47]. The relative humidity was maintained at 60–80 ​%, and the photoperiod was set to 11 ​h (7:00–18:00) with photosynthetically active radiation (PAR) of 800 ​μmol ​m^−2^ ​s^−1^. The experimental plants were placed in and removed from the growth chamber at 8:00 a.m. After acclimatization for 1 ​h in the laboratory, chlorophyll fluorescence imaging, hyperspectral imaging, light response curves, relative electrical conductivity, and chlorophyll content were measured sequentially. All parameters were measured on the same leaf to minimize unanticipated bias due to physiological differences among leaves.

### 2.4 Data collection

#### 2.4.1 Maximum net photosynthetic rate

The light response curves of the strawberry leaves were measured using a Li-6400xt portable photosynthesis system (LI-COR, USA). Healthy, fully expanded functional leaves located on the outer canopy with minimal inclination were selected as the observation targets, and the same leaves were used for other physiological measurements. The light gradient in the instrument's analysis chamber was set to 1600, 1400, 1200, 1000, 800, 600, 400, 200, 100, 50, 20, and 0 ​μmol ​m^−2^ ​s^−1^, with other parameters following the settings of Jiang et al. [48]. The system automatically recorded the net photosynthetic rate (P_n_) of the strawberry leaves under different light intensities, and the maximum net photosynthetic rate (P_max_) was obtained by fitting the light response curves using a mechanistic model [49].

#### 2.4.2 Relative electrolyte conductivity

The leaves were first cleaned with deionized water, and 0.2 ​g of leaf tissue, avoiding the midrib, was excised. The leaf samples were cut into small pieces (d ​≤ ​5 ​mm) and placed into a 25 ​mL Erlenmeyer flask with 20 ​mL of deionized water. After a 20-min vacuum treatment to ensure that the leaf fragments settled at the bottom, the conductivity (C1) of the mixture was measured using a DDS-307 ​A conductivity meter (INESA, China). The flask was then subjected to a boiling water bath for 20 ​min, and after the mixture cooled to room temperature (24 ​°C), the conductivity (C2) was measured again. The relative electrolyte conductivity was calculated using Equation (1).

(1) REC=C1/C2

#### 2.4.3 Chlorophyll content

For the chlorophyll content measurement, 0.2 ​g of leaf fragments, prepared in the same manner as those used for the REC measurement, were placed in a 30 ​mL amber glass bottle with 25 ​mL of 95 ​% ethanol (analytical grade) and sealed for 48 ​h in the dark. After the leaf fragments had been completely decolorized, the absorbances at 649 ​nm and 665 ​nm were measured using a UV-1800 UV spectrophotometer (Shimadzu, JP). The total chlorophyll content of the strawberry leaves was calculated using Equations (2), (3), (4), (5).

(2) $$c_{a}=13.95A_{665}-6.88A_{649}$$

(3) $$c_{b}=24.96A_{649}-7.32A_{665}$$

(4) $$c_{a+b}=c_{a}+c_{b}$$

(5) $$C_{a+b}=\left(c_{a+b}\times V\times D\right)/m$$

In these equations, c_a_, c_b_, and c_a ​+ ​b_ represent the concentrations of chlorophyll *a*, chlorophyll *b*, and total chlorophyll, respectively. C_a ​+ ​b_ represents the total chlorophyll content (mg g^−1^), V represents the volume of the extraction solution, D represents the dilution factor, and m represents the fresh weight of the sample.

#### 2.4.4 Chlorophyll fluorescence imaging

Chlorophyll fluorescence imaging was conducted using the PlantExplorer PRO multifunctional plant photosynthesis phenotyping system (PhenoVation, NL). This system is equipped with a 12-megapixel CMOS image sensor covering a spectral range of 350–1000 ​nm, a 12-bit filter wheel including fluorescence filters, and a six-channel LED light source. The light source spectrum included 450 ​nm blue light, 660 ​nm red light, 730 ​nm far-red light, and 3000 ​K white light. After 30 ​min of dark adaptation, the strawberry plants were placed in the observation chamber, and Pulse Amplitude Modulated (PAM) and Relative Fluorescence Decay (Rfd) measurement modules were selected. The instrument automatically executed the measurement protocol, with a saturating light pulse duration of 10 ​s and a total measurement time of 300 ​s. The distance between the strawberry canopy and the light source/camera was approximately 40 ​cm, providing an excitation light intensity of approximately 5000 ​μmol ​m^−2^ ​s^−1^ and an actinic light intensity of approximately 600 ​μmol ​m^−2^ ​s^−1^.

To facilitate subsequent spatial variability analysis, the Fo, Fm, Fo’, Fm’, and Fs grayscale images obtained from the instrument were processed to remove abnormal pixels. Using formulas for chlorophyll fluorescence induction kinetics, we generated phenotypic raster images for eight classical fluorescence parameters: Fv/Fm, NPQ, qP, qL, qN, Y(II), Y(NPQ), and Y(NO) [50,51].

#### 2.4.5 Hyperspectral imaging

Hyperspectral reflectance images of strawberry leaves were captured via a SOC710 portable hyperspectral imager (Surface Optics Corporation, USA). Two 75 ​W PAR30 halogen lamps (OSRAM, Germany) served as artificial light sources, with a reference gray card placed near the target leaf to ensure similar lighting conditions. The working spectral range of the imager was 375.88–1039.19 ​nm, and the 256-band mode with a spectral resolution of 2.6 ​nm was used for imaging. The imager was positioned approximately 40 ​cm directly above the strawberry canopy, with a pixel exposure time of 19 ​ms.

The acquired digital signal cube was subjected to wavelength calibration, dark pixel radiation calibration, and NIST reference plate correction, followed by reflectance cube conversion. To mitigate the effects of non-uniform light field intensity distributions from artificial light sources and considering that the characteristic spectral bands of different plants or targets may not align perfectly, we focused on the average reflectance images within four specific spectral bands: blue (475–485 ​nm), green (545–555 ​nm), red (645–655 ​nm), and near-infrared (780–790 ​nm). Using these bands, we constructed normalized difference index images, similar to the NDVI, between each pair of bands. The strawberry reflectance spectral feature indices developed in this study are listed in Table 2.

**Table 2:** Reflectance spectral indices used in this study.

Index	Calculation Formula^a^
NDVI	(NIR−R)/(NIR+R)
NDVInb	(NIR−B)/(NIR+B)
NDVIng	(NIR−G)/(NIR+G)
NDVIgb	(G−B)/(G+B)
NDVIgr	(G−R)/(G+R)

a NIR, R, G, and B represent reflectance images in the near-infrared, red, green, and blue spectral bands, respectively.

#### 2.4.6 Relative negative accumulated temperature

In this study, Relative Negative Accumulated Temperature (RNAT) is defined as the integral of the hourly temperature difference between the low-temperature treatment group and the control group over the duration of the treatment. As such, the RNAT for the control group was 0, whereas the RNAT for the low-temperature treatment groups increased monotonically with decreasing temperature and increasing treatment duration. RNAT was calculated using Equation (6).

(6) $$\text{RNAT}=\sum_{t=0}^{n}T_{\text{CK}}\left(t\right)-T_{\text{LT}}\left(t\right)$$

where t represents the current time, n represents the total duration of the low-temperature stress in hours, T_CK_(t) represents the temperature in the control group at time t, and T_LT_(t) represents the temperature in the low-temperature treatment group at time t.

### 2.5 Phenotypic spatial variability feature extraction

#### 2.5.1 Extraction of phenotypic features across different dimensions and directions

To elucidate the typical spatial variability characteristics of the strawberry leaf phenotypes under low-temperature stress and reveal the spatial evolution trends of cold damage in the leaves, this study extracted phenotypic features in both one-dimensional (1D) transverse and two-dimensional (2D) planar dimensions. The 1D transverse features included two sub-features: one parallel to the main vein and the other perpendicular to it. The 1D transverse features spanned the leaf along a specific direction, with both endpoints located within the leaf. The 2D planar feature covered an irregular, near-circular area that generally encompassed the entire leaf. Detailed information about the strawberry leaf phenotypic features extracted across different dimensions and directions is provided in Table 3.

**Table 3:** Phenotypic features of the strawberry leaves extracted in this study.

Phenotypic Feature	1D - Parallel to Main Vein	1D - Perpendicular to Main Vein	2D - Region Feature
Feature Marker	1D - Parallel	1D - Perpendicula	2D - Region
Definition	A non-overlapping line segment parallel to the main vein, spanning the leaf with both endpoints inside.	A perpendicular segment crossing the main vein at the leaf's widest point, with both endpoints inside.	An irregular near-circular area covering most of the leaf, enclosed within it.
Extraction Example			

#### 2.5.2 Calculation of spatial variability feature parameters

To analyze the changes in the phenotypic features of the strawberry leaves under low-temperature stress, this study utilized eight statistical features (Mean, Variance, Coefficient of Variation, Interquartile Range, Skewness, Kurtosis, Inertia, and Autocorrelation) and eight structural features (Shannon Entropy, Laplace Entropy, Contrast, Angular Second Moment, Texture Entropy, Homogeneity, Inertia Moment, and Small Gradient Advantage) as spatial variability feature parameters. These parameters were used to assess variations in numerical values, spatial distributions, and texture structures across different dimensions and directions.

Importantly, for the one-dimensional transverse features, the Contrast, Energy, Texture Entropy, Homogeneity, Inertia Moment, and Small Gradient Advantage were calculated by stacking and repeating the one-dimensional matrix in the vertical direction to form a square matrix with equal rows and columns. Detailed information about the spatial variability feature parameters used for this study is provided in Table 4.

**Table 4:** Spatial variability feature parameters used in this study.

Feature Type	Parameter	Calculation Formula	Significance	Notes
Statistical Features	Mean	$A=\frac{1}{N}\sum_{i=1}^{N}x_{i}$	Overall level of the data	i: the ith pixel; N: the total number of pixels; X_i_: value of the ith pixel; X_i+1_: value of the (i+1)th pixel; Q1: lower quartile; Q3: upper quartile.
	Variance	$\text{VAR}=\frac{1}{N}\sum_{i=1}^{N}\left(x_{i}-A\right)$	Degree of deviation from the mean	
	Coefficient of Variation	$\text{COV}=\frac{\sigma }{A}\times 100\%$	Relative dispersion of the data	
	Interquartile Range	IQR=Q3−Q1	Range of the middle 50 ​% of the data	
	Skewness	$\text{SKE}=\frac{1}{N}{\sum_{i=1}^{N}\left(\frac{x_{i}-A}{\sigma }\right)}^{3}$	Symmetry of the data distribution relative to the mean	
	Kurtosis	$\text{KUR}=\frac{1}{N}{\sum_{i=1}^{N}\left(\frac{x_{i}-A}{\sigma }\right)}^{4}-3$	Steepness of the data relative to a normal distribution	
	Inertia	$\text{INE}={\sum_{i=2}^{N}\left(\text{xi}-x_{i-1}\right)}^{2}$	Intensity or volatility of the changes between data points	
	Autocorrelation	$\text{ACOR}=\frac{\sum_{i=1}^{N-1}\left(x_{i}-A\right)\left(x_{i+1}-A\right)}{\sum_{i=1}^{N}{\left(x_{i}-A\right)}^{2}}$	Linear relationship between data points and their subsequent points	
Structural Features	Shannon Entropy	$\text{SENT}=-\sum_{i=1}^{N}p_{i}\;\log_{2}\left(p_{i}+\varepsilon \right)$	Complexity or disorder of the information	p_i_: probability of the ith pixel value; *ε*: small constant (10^−10^) to avoid zero in logarithmics; g_i_:the ith gradient value; N_g_: total number of gradients.
	Laplace Entropy	. $\text{LENT}=-\sum_{i=1}^{\text{Ng}}g_{i}\;\log_{2}\left(\left|g_{i}\right|+\varepsilon \right)$.	Distribution of the data change rates and complexity of information	
	Contrast	$\text{CON}=\sum_{i,j}{\left(i-j\right)}^{2}P\left(i,j\right)$	Degree of difference between the grayscale levels in the image	i, j: indices of the grayscale values; P (i,j): probability at (i,j) in the gray-level co-occurrence matrix (GLCM); *ε*: small constant (10^−10^).
	Angular Second Moment	$\text{ASM}=\sum_{i,j}P{\left(i,j\right)}^{2}$	Concentration of the grayscale distribution in the image	
	Texture Entropy	$\text{TENT}=-\sum_{i,j}P\left(i,j\right)\log_{2}\left(P\left(i,j\right)+\varepsilon \right)$	Disorder of the image texture	
	Homogeneity	$\mathrm{HOM}=\sum_{i,j}\frac{P\left(i,j\right)}{1+{\left(i-j\right)}^{2}}$	Uniformity or consistency of the grayscale distribution in the image	
	Inertia Moment	$\text{INEM}=\sum_{i,j}\left(i^{2}+j^{2}\right)P\left(i,j\right)$	Distribution of the grayscale differences in the image	
	Small Gradient Advantage	$\text{SGA}=\sum_{i=1}^{M}\sum_{j=1}^{N}G_{\text{glcm}}\left(i,j\right)$	Frequency of the small grayscale differences (small gradients) in the image	M, N: number of rows and columns in the GLCM; G_glcm_ (i,j): value at (i,j) in the GLCM gradient matrix.

#### 2.5.3 Feature selection method

Mutual Information (MI) is a measure used to evaluate the dependency between two variables. In this study, MI was employed to identify the key multi-source phenotypic spatial variability features most dependent on the strawberry photosynthetic physiological parameters. The relationships between photosynthetic physiology and spatial variability features under the varying low-temperature gradients and durations were not necessarily linear or even monotonic. Unlike traditional correlation measures, MI can capture both linear and nonlinear relationships, making it particularly advantageous in feature selection processes. MI has been successfully applied in various fields, including medical screening, motion recognition, and meteorological data selection [[52], [53], [54]]. The MI quantifies the amount of information shared between two random variables, and its formal definition is given by Equation (7).

(7) $$\text{MI}\left(X;Y\right)=\sum_{x\in X}\sum_{y\in Y}p\left(x,y\right)\log\frac{p\left(x,y\right)}{p\left(x\right)p\left(y\right)}$$

where p (x,y) represents the joint probability distribution of variables x and y and where p(x) and p(y) are the marginal probability distributions of x and y, respectively.

### 2.6 Modeling methods and model interpretation

#### 2.6.1 Tree-based ensemble machine learning models

For modeling the monitoring and risk prediction of low-temperature damage in strawberries, tree-based ensemble machine learning models offer a superior choice over neural network models, especially when the scale of the experimental data was considered. These models not only mitigate the risk of overfitting but also provide enhanced interpretability [55]. Tree-based ensemble models use decision trees as their fundamental building blocks, improving predictive performance by aggregating multiple trees. Among these, XGBoost (XGB), AdaBoost (AB), and Random Forest (RF) represent different strategies within tree-based ensemble algorithms [56]. XGB and AB are Boosting methods where trees are sequentially dependent; new trees are constructed progressively based on the performance of previous trees. The key difference lies in their optimization: XGB minimizes the loss function's gradient, whereas AB adjusts sample weights to focus on difficult-to-classify instances. RF, on the other hand, is a Bagging method where trees are independently built in parallel, making it less sensitive to noise and outliers. In this study, the key model parameters, “n_estimators” and “max_depth,” were optimized according to the input data type and algorithm, with a 5-fold cross-validation procedure. The training and validation samples were completely independent, with a 5:1 ratio.

#### 2.6.2 Model interpretation algorithms

Explainable Artificial Intelligence (XAI) has emerged as one of the most prominent topics in machine learning research. Among various XAI techniques, SHapley Additive exPlanations (SHAP) has become the standard for model interpretation, providing insights into the mechanisms within the “black box” of machine learning models [57,58]. The core idea of the SHAP algorithm is rooted in Shapley values, a concept from game theory [59]. SHAP values quantify the contribution of each feature to the prediction, including both the magnitude and direction of the impact, as well as the interactions between features. This makes SHAP an invaluable tool for understanding the key decision-making processes in models. In this study, after the models for monitoring and predicting low-temperature damage in strawberries were developed, SHAP was used to analyze the influence of critical multi-source spatial variation features on PPPI and RNAT, as well as to explore the interactions and dependencies among key phenotypic features.

### 2.7 Statistical analysis and software implementation

SPSS 24 (IBM, USA) was used for one-way analysis of variance (ANOVA) and Duncan's multiple range test (P ​= ​0.05). Principal component analysis (PCA) of P_max_, REC, and Chl_a ​+ ​b_, along with the construction of the PPPI, were carried out using Origin 2021 (OriginLab, USA). The accuracy of the low-temperature damage monitoring and risk prediction models, which combine multi-source phenotypic spatial variation features, was evaluated using the coefficient of determination (R^2^) and root mean square error (RMSE). The formulas for R^2^ and RMSE are provided in Equations (8), (9), respectively.

(8) $$R^{2}=1-\frac{\sum_{i=1}^{n}{\left(y_{i}-{\hat{y}}_{i}\right)}^{2}}{\sum_{i=1}^{n}{\left(y_{i}-\bar{y}\right)}^{2}}$$

(9) $$\text{RMSE}=\sqrt{\frac{\sum_{i}^{n}{\left(y_{i}^{ˆ}-y_{i}\right)}^{2}}{n}}$$

where $y_{i}$ represents the actual value of the ith sample, $\hat{y_{i}}$ represents the predicted value of the ith sample, $\bar{y}$ is the mean of all actual sample values, and n is the total number of samples.

The phenotypic prediction performance of the strawberry low-temperature damage risk levels, delineated based on the low-temperature risk index, was evaluated using a confusion matrix combined with accuracy (ACC) and the Kappa coefficient (Kappa). The formulas for the ACC and Kappa are given in Equations (10), (11), (12), (13).

(10) $$\text{ACC}=P_{o}\times 100\%$$

(11) $$\text{Kappa}=\frac{P_{o}-P_{e}}{1-P_{e}}$$

(12) $$P_{o}=\frac{\sum_{i=1}^{C}T_{i}}{n}$$

(13) $$P_{e}=\frac{\sum_{i=1}^{C}a_{i}\times b_{i}}{n^{2}}$$

where $p_{o}$ is the overall classification accuracy, $p_{2}$ is the expected classification accuracy, n is the total number of samples, C is the total number of categories, $T_{i}$ is the number of correctly classified samples for each category, a_1_, a_2_, …, a_C_ represent the actual number of samples in each category, and b_1_, b_2_, …, b_C_ represent the predicted number of samples in each category.

Chlorophyll fluorescence induction kinetics parameter imaging, hyperspectral imaging band extraction and calculation, phenotypic distribution feature extraction, spatial variation feature parameter calculation, and MI analysis were implemented via MATLAB R2021b. Model parameter selection, the PPPI phenotypic inversion model, the RNAT phenotypic inversion model, and the SHAP explainable model were implemented using Python 3.8.

## 3 Results

### 3.1 Effects of cold stress on strawberry photosynthetic physiology

#### 3.1.1 Impact of cold stress on photosynthetic parameters

**Fig. 3:**
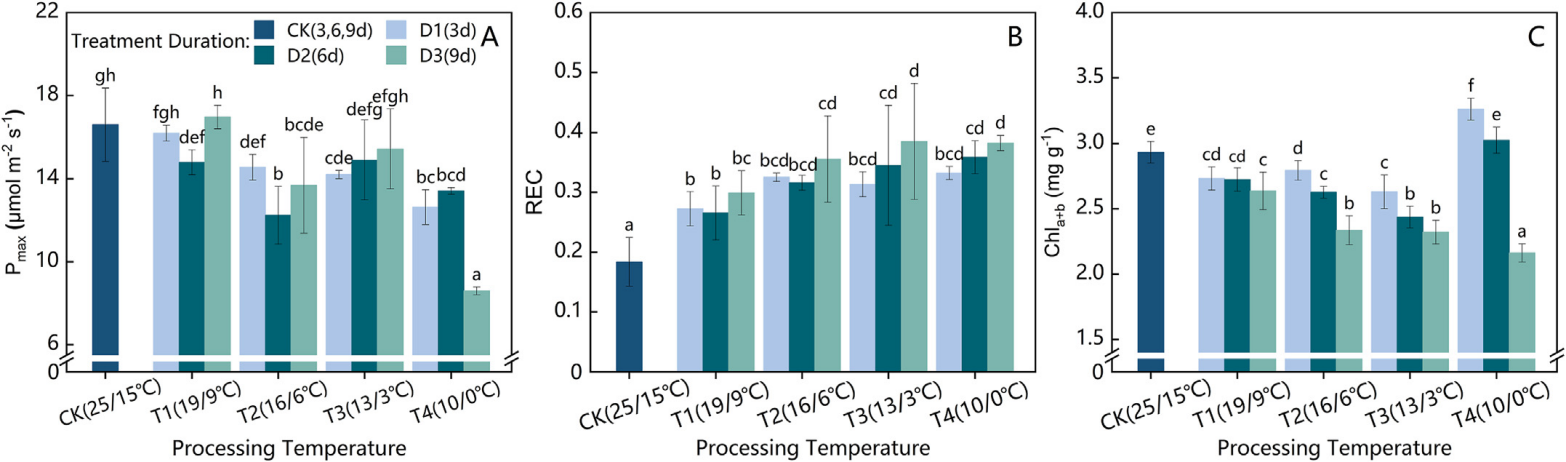
Variations in the photosynthetic physiological parameters of the strawberry leaves under the different cold stress gradients and durations: (A) maximum photosynthetic rate, (B) relative electrolyte conductivity, and (C) total chlorophyll content.

As shown in Fig. 3(A), under the T1 (19/9 ​°C) and T2 (16/6 ​°C) cold stress gradients, the maximum net photosynthetic rate (P_max_) of the strawberry plants initially decreased but then increased with prolonged stress duration, reaching its lowest point at D2 (6 days). During the D1 (3 days) and D2 stress periods, P_max_ under T2 was consistently lower than that under T1. By D3 (9 days), P_max_ under T1 had recovered to a level similar to that of the control (CK) and T1D1 treatments, whereas Pmax under T2D3 remained slightly lower than that under T2D1, although not significantly, with a reduction of 12.26 ​% compared with that under CK. Under the T3 (13/3 ​°C) and T4 (10/0 ​°C) gradients, P_max_ decreased to its lowest value at D1 and then began to increase. P_max_ under T3 continued to rise from D1 to D3, whereas, under T4, it slightly increased from D1 to D2 before dropping sharply to 8.601 ​μmol ​m^−2^ ​s^−1^ at T4D3, representing decreases of 35.89 ​% and 48.18 ​% compared with those under the T4D2 and CK treatments, respectively.

As the treatment temperature decreased and the duration increased, the relative electrolyte conductivity (REC) of the strawberry leaf tissues generally initially increased but then plateaued (Fig. 3(B)). Under short-term D1 stress, the REC was relatively similar under the T2, T3, and T4 cold environments, with an average value of 0.324. Under the T3 and T4 treatments, the REC remained stable between D2 and D3, with mean values of 0.352 and 0.384, respectively, which were 91.29 ​% and 108.66 ​% greater than that of the CK.

Fig. 3(C) shows that the total chlorophyll content (Chl_a ​+ ​b_) in the strawberry leaves generally decreased with increasing duration of stress under the T1 to T3 treatments. Under T1 conditions, Chl_a ​+ ​b_ did not significantly differ over the duration from D1 to D3. However, under the T2 treatment, Chl_a ​+ ​b_ significantly decreased within each 3-day interval as the stress duration increased. The Chl_a ​+ ​b_ levels under T2D3 and T3D3 were similar, with an average of 2.329 ​mg ​g^−1^, which was 79.4 ​% that of the CK. Under T4D1, the Chl_a ​+ ​b_ was 3.26 ​mg ​g^−1^, 7.77 ​% and 11.15 ​% higher than that under T4D2 and CK, respectively. However, T4D3 presented the lowest Chl_a ​+ ​b_ level across all the treatments, at 2.163 ​mg ​g^−1^, representing a 26.26 ​% decrease compared with that of the CK.

#### 3.1.2 Construction of the Photosynthetic Physiological Potential Index

**Fig. 4:**
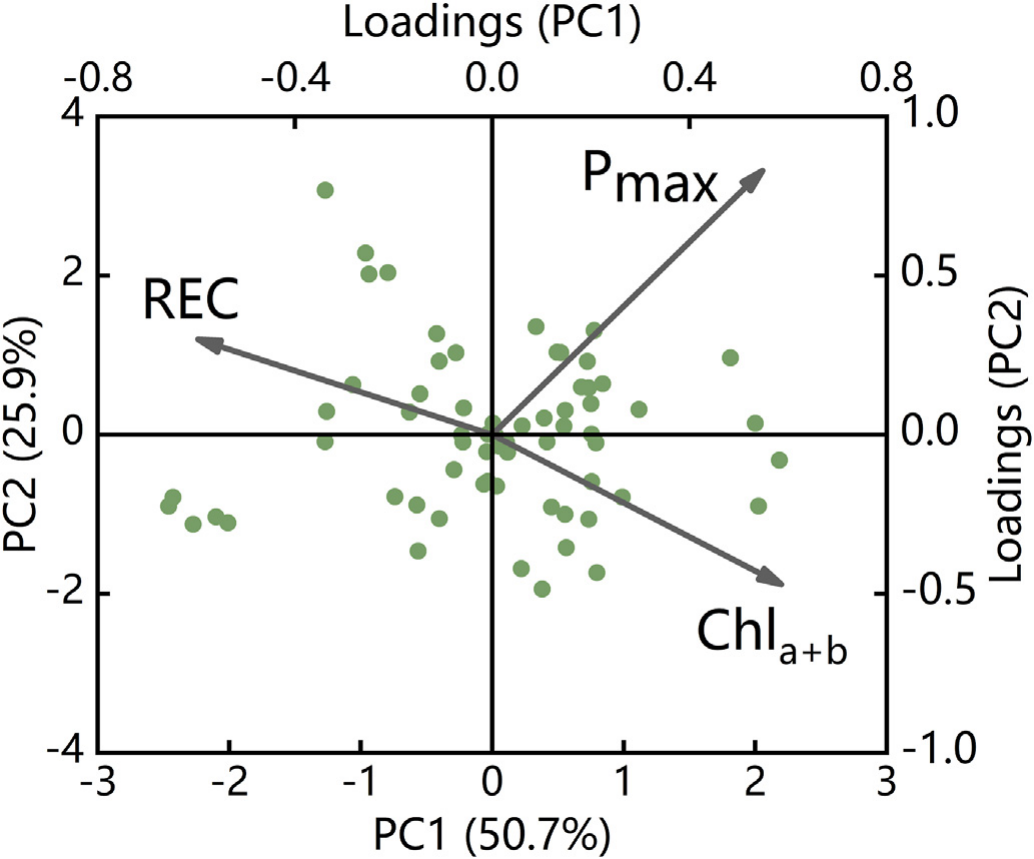
PCA scores and loadings of P_max_, REC, and Chl_a ​+ ​b_ under the different degrees of cold stress on PC1 and PC2.

To overcome the limitations of relying on a single data type and to more comprehensively evaluate the changes in strawberry photosynthetic physiological activity under the varying cold stress gradients and durations, a principal component analysis (PCA) was performed on three photosynthetic physiological parameters: P_max_, REC, and Chl_a ​+ ​b_. These parameters, each with distinct physiological significance, were used to construct the Photosynthetic Physiological Potential Index (PPPI) based on their scores and loadings. The PCA scores and loadings for P_max_, REC, and Chl_a ​+ ​b_ on the first two principal components (PC1 and PC2) are illustrated in Fig. 4.

The cumulative variance contribution rates of PC1 and PC2 for the three photosynthetic physiological parameters were 50.7 ​% and 25.9 ​%, respectively, which collectively explained 76.6 ​% of the variance. The absolute value of the loading for REC PC1 was the highest at 0.596, acting in the opposite direction to P_max_ and Chl_a ​+ ​b_. For PC2, P_max_ and REC had positive loadings, whereas Chl_a ​+ ​b_ contributed negatively, with P_max_ showing the highest absolute loading value of 0.83, significantly surpassing the other two parameters.

Generally, a cumulative variance contribution rate of 85 ​% or higher is required to effectively represent the majority of the information in the data [60]. In the PCA of P_max_, REC, and Chl_a ​+ ​b_, the cumulative variance contribution rates of PC1, PC2, and PC3 reached 100 ​%, indicating that these three principal components could explain all the variance in the data. The loadings of P_max_, REC, and Chl_a ​+ ​b_ on PC1, PC2, and PC3, along with the eigenvalues of each principal component, are presented in Table 5.

**Table 5:** Principal component analysis results for P_max_, REC, and Ch_la ​+ ​b_ under the different degrees of cold stress.

Principal Components		PC1	PC2	PC3
Eigenvalue		1.52	0.778	0.703
Variance Contribution (%)		50.657	25.926	23.417
Cumulative Variance Contribution (%)		50.657	75.583	100
Loadings	P_max_	0.548	0.83	0.105
	REC	−0.596	0.3	0.745
	Chl_a ​+ ​b_	0.586	−0.471	0.659

Using the loadings of the three photosynthetic physiological parameters on each principal component, as provided in Table 5, and their respective variance contribution rates, the scores for PC1, PC2, and PC3 were calculated. These scores were then weighted by the variance contribution rates of each principal component and summed to obtain the Photosynthetic Physiological Potential Index (PPPI). The formulas for calculating the PPPI are provided in Table 6. A higher PPPI indicates better photosynthetic physiological potential in strawberry plants, whereas a PPPI close to zero suggests poor potential, implying that the plants are under significant stress.

**Table 6:** Formulas for calculating PC1, PC2, and PC3 scores and the Photosynthetic Physiological Potential Index based on P_max_, REC, and Chl_a ​+ ​b_.

Variables	Formulas
PC1 Score	$S_{1}=0.548\times P_{\max}-0.596\times \text{REC}+0.586\times {\text{Chl}}_{a+b}$
PC2 Score	$S_{2}=0.83\times P_{\max}+0.3\times \text{REC}-0.471\times {\text{Chl}}_{a+b}$
PC3 Score	$S_{3}=0.105\times P_{\max}+0.745\times \text{REC}+0.659\times {\text{Chl}}_{a+b}$
Photosynthetic Physiological Potential Index	$\text{PPPI}=0.507\times S_{1}+0.259\times S_{2}+0.234\times S_{3}-5$

The levels of PPPI in the strawberry plants under the different degrees of cold stress and varying durations are presented in Table 7. After 3 days of stress, the PPPI of the strawberry plants decreased as the treatment temperature decreased. After 6 days at 16/6 ​°C, the PPPI decreased significantly by 30.4 ​% compared with that at 3 days, reaching 47.37 ​% of the CK value, and recovered to a level similar to that at 3 days after 9 days of stress. At 10/0 ​°C for 6 days, the PPPI increased by 12.61 ​% compared with that at 3 days, but when the stress duration increased to 9 days, the PPPI dramatically decreased to nearly zero. Over the 9-day stress period, the PPPI of the strawberry plants consistently decreased as the temperature decreased.

**Table 7:** Levels of the Photosynthetic Physiological Potential Index in the strawberry plants under the different degrees of cold stress and varying stress durations.

PPPI (CK: 4.545 ​± ​0.824)	Duration of stress		
Daily Maximum Temperature/Daily Minimum Temperature	3d	6d	9d
19/9 ​°C	4.268 ​± ​0.178	3.539 ​± ​0.29	3.699 ​± ​0.374
16/6 ​°C	3.437 ​± ​0.296	2.392 ​± ​0.464	3.352 ​± ​0.63
13/3 ​°C	3.2 ​± ​0.104	2.876 ​± ​0.385	2.025 ​± ​0.26
10/0 ​°C	2.593 ​± ​0.387	2.92 ​± ​0.101	0.142 ​± ​0.094

### 3.2 Extraction of key features with phenotypic spatial variability in strawberries

#### 3.2.1 Mutual information analysis between phenotypic spatial variability and photosynthetic physiological parameters

**Fig. 5:**
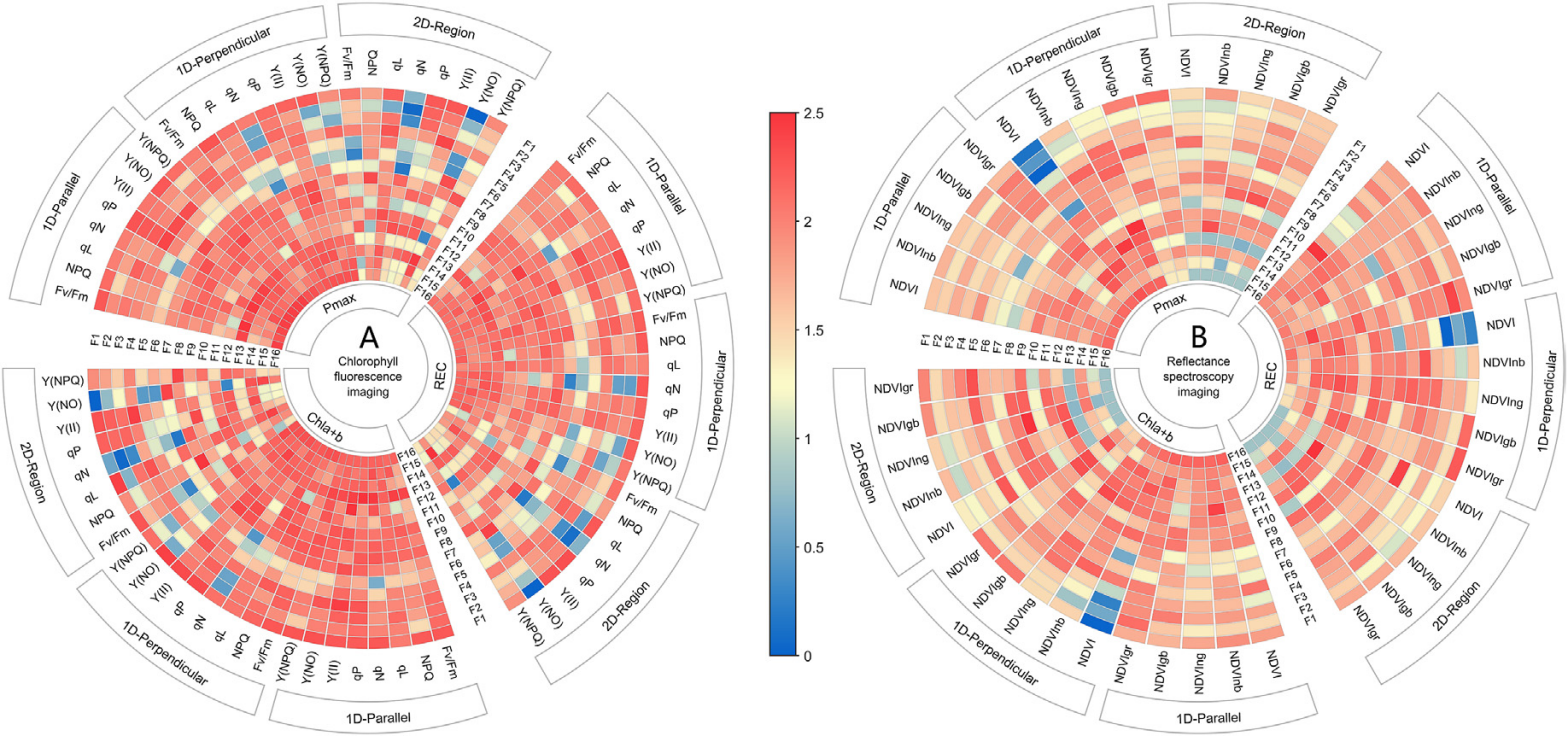
Mutual information values between the phenotypic spatial variability features and P_max_, REC, and Chl_a ​+ ​b_ in the strawberries: F1 to F8 represent statistical features—A, VAR, COV, IQR, SKE, KUR, INE, ACOR, and F9 to F16 represent structural features—SENT, LENT, CON, ASM, TENT, HOM, INEM, SGA.

To elucidate the specific photosynthetic physiological significance embedded in strawberry phenotypic traits distributed across various spatial dimensions, as captured through chlorophyll fluorescence induction kinetics and hyperspectral index images, we conducted spatial variability feature analysis. This analysis encompassed eight statistical features and eight structural features derived from the image analysis metrics described in Table 4 for each data source across different dimensions and orientations. The mutual information (MI) between these phenotypic spatial variability features and the photosynthetic parameters P_max_, REC, and Chl_a ​+ ​b_ was analyzed, and the MI value heatmap is presented in Fig. 5. The MI value reflects the dependency between two parameters: the larger the value is, the stronger the dependency, whereas values close to zero indicate a weak relationship between the parameters.

As shown in Fig. 5(A), for the phenotypic traits related to chlorophyll fluorescence induction kinetics, the spatial variability features in the 1D-Parallel direction (parallel to the main leaf vein) generally presented relatively high MI values with P_max_, REC, and Chl_a ​+ ​b_. In contrast, the spatial variability features across the 2D-Region (the leaf surface area) predominantly displayed mid-to-low MI values, whereas those in the 1D-Perpendicular direction (perpendicular to the main leaf vein) presented intermediate MI values. Notably, in the 1D-Parallel direction, the texture entropy (TENT) of the fluorescence parameters and the interquartile range (IQR) of qP, Y(II), and Y(NO) presented high MI values with P_max_, REC, and Chl_a ​+ ​b_. In the 2D-Region dimension, the autocorrelation (ACOR) of NPQ and Y(NPQ), as well as the inertia (INE) of Y(NO), also maintained high MI values with the three photosynthetic parameters. Additionally, the spatial variability features of Y(NO) and qN were characterized by both high and low MI values.

Fig. 5(B) displays the MI analysis results between the hyperspectral index phenotypic spatial variability features and the three photosynthetic parameters, revealing that the overall MI values were lower than those of the chlorophyll fluorescence phenotypic spatial variability features. The phenotypic spatial variability features with greater dependency on P_max_ were primarily concentrated in the contrast (CON), angular second moment (ASM), and texture entropy (TENT) of the NDVI, NDVInb, and NDVIng in the 1D-Perpendicular direction. Although the spatial variability features of NDVIng in this direction generally showed good MI dependency, relatively high values were also observed in the Laplacian entropy (LENT) of the NDVI in the 1D-Parallel direction and the ASM of NDVInb in the 2D-Region. Similarly, the spatial variability features with relatively high MI values with Chl_a ​+ ​b_ were discretely distributed, including the ASM of NDVInb in the 1D-Parallel direction and the Shannon entropy (SENT) of NDVIng in the 2D-Region. Additionally, the SENT, CON, and ASM of the hyperspectral index phenotypic features across the respective spectral bands demonstrated a strong dependency relationship with P_max_, REC, and Chl_a ​+ ​b_.

The top two phenotypic spatial variation features, ranked by MI values with each of the three photosynthetic physiological parameters, were identified across different dimensions and distribution directions (See Table 8). These features were defined as typical features, and their corresponding phenotypic parameters, spatial variation feature types, and MI values are presented in Table 7. As shown in the table, among the 16 phenotypic spatial variation features, ASM was the most frequently identified typical feature, appearing seven times, followed by A, which appeared five times. Other features, such as TENT, SENT, HOM, and SGA, each appeared four times.

**Table 8:** The typical spatial variation features in phenotype across the different dimensions and directions related to the strawberry photosynthetic physiological parameters.

Typical Spatial Variation Features in Phenotype		P_max_		REC		Chl_a ​+ ​b_	
		Feature	MI	Feature	MI	Feature	MI
Chlorophyll Fluorescence Imaging	1D-Parallel	NPQ/TENT^a^	2.208	qN/TENT	2.049	qP/TENT	2.442
		Y (Ⅱ)/SGA	2.139	Y (Ⅱ)/A	1.964	Y (Ⅱ)/ASM	2.441
	1D-Perpendicular	Y (Ⅱ)/SENT	2.079	qP/HOM	1.931	Y(NO)/A	2.362
		NPQ/SGA	2.045	qN/A	1.922	Y (Ⅱ)/HOM	2.339
	2D-Region	Y(NO)/HOM	2.168	Y(NO)/INEM	2.118	NPQ/ACOR	2.424
		Y (Ⅱ)/IQR	2.104	qP/A	1.969	qL/A	2.363
Hyperspectral Imaging	1D-Parallel	NDVIgr/TENT	2.102	NDVI/LENT	2.047	NDVInb/ASM	2.375
		NDVIgb/SGA	2.018	NDVIgr/HOM	1.981	NDVI/SGA	2.271
	1D-Perpendicular	NDVIng/CON	2.207	NDVIng/ASM	1.95	NDVIng/CON	2.345
		NDVInb/ASM	2.18	NDVInb/SKE	1.932	NDVIgr/ASM	2.335
	2D-Region	NDVInb/ASM	2.099	NDVInb/ASM	2.057	NDVIng/SENT	2.43
		NDVIgb/SENT	2.092	NDVI/KUR	2.001	NDVIgr/SENT	2.203

a The shorthand “X/Y” denotes the spatial variation feature (Y) of the phenotypic imaging parameter (X).

To further refine these typical phenotypic spatial variation features, the two features with the highest dependency on each photosynthetic physiological parameter were selected as the key features. These key features were used as critical input variables for modeling strawberry cold stress monitoring and risk early warning. The six key features identified were: TENT of NPQ in the 1D-Parallel direction (NPQ/1D-Parallel/TENT), CON of NDVIng in the 1D-Perpendicular direction (NDVIng/1D-Perpendicular/CON), Inertia Moment (INEM) of Y(NO) in the 2D-Region (Y(NO)/2D-Region/INEM), ASM of NDVInb in the 2D-Region (NDVInb/2D-Region/ASM), TENT of qP in the 1D-Parallel direction (qP/1D-Parallel/TENT), and ASM of Y (Ⅱ) in the 1D-Parallel direction (Y (Ⅱ)/1D-Parallel/ASM).

#### 3.2.2 Impact of cold stress on the variability in key phenotypic spatial features of strawberries

**Fig. 6:**
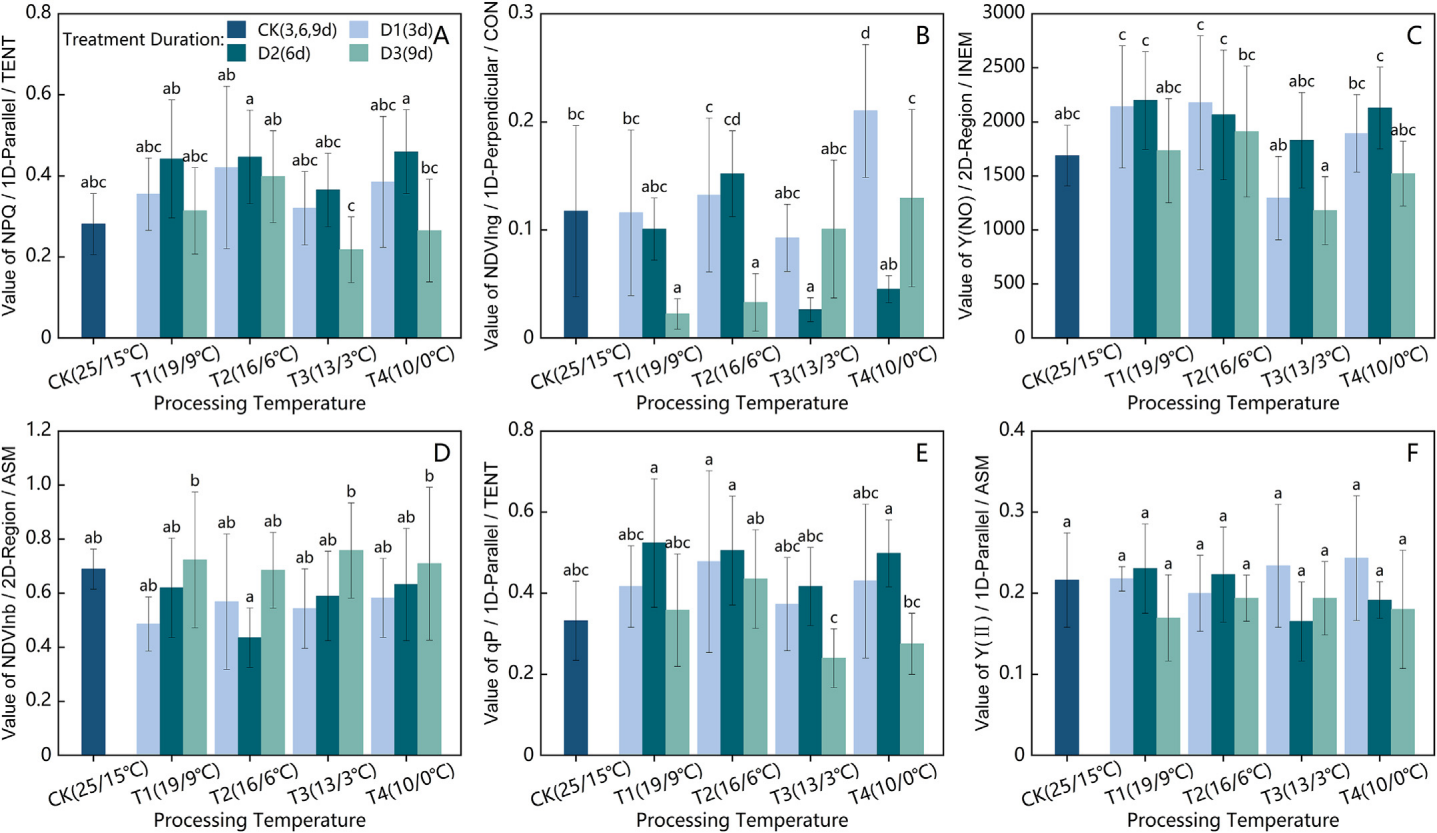
Variations in the key phenotypic spatial variability features of strawberry plants under different cold stress gradients and durations: (A) Texture Entropy of NPQ along the 1D-Parallel direction; (B) Contrast of NDVIng along the 1D-Perpendicular direction; (C) Inertia Moment of Y(NO) in the 2D-Region; (D) Angular Second Moment of NDVInb in the 2D-Region; (E) Texture Entropy of qP along the 1D-Parallel direction; (F) Angular Second Moment of Y (Ⅱ) along the 1D-Parallel direction.

Fig. 6 shows the changes in six key phenotypic spatial variability features of strawberry plants under different temperature gradients and durations of cold stress. As shown in Fig. 6(A), NPQ/1D-Parallel/TENT initially increased but then decreased with prolonged stress exposure at all the temperatures. After treatment durations D1 and D2, TENT increased relative to CK at all temperatures, with the increase being more pronounced under D2. Under the D3 treatment, TENT peaked at 0.398 ​at T2 and then significantly declined as the temperature decreased. Fig. 6(B) shows that NDVIng/1D-Perpendicular/CON remained stable during the first 6 days at T1 and T2, with CON slightly higher at T2 than at T1. However, at D3, the CON decreased sharply to 18.88 ​% and 28.03 ​% of the CK value at T1 and T2, respectively. At T3 and T4, CON also decreased significantly during D2, dropping to 22.29 ​% and 38.38 ​% of CK at T3D2 and T4D2, respectively, before rebounding to CK levels by D3. Additionally, CON increased to 0.21 under T4D1, which was significantly greater than that in the other treatment groups. Fig. 6(C) shows that Y(NO)/2D-Region/INEM initially increases and then decreases with prolonged stress exposure at T1 and T2, with no significant differences between D1 and D2. At T3D2 and T4D2, the Y(NO)/2D-Region/INEM levels were similar to those at T1 and T2 during the same treatment period but significantly lower than T1 and T2 at D1 and D3. As depicted in Fig. 6(D), NDVInb/2D-Region/ASM increased with the duration of stress at T1, T3, and T4 and increased as the temperature decreased from T1 to T4 under the D1 treatment, although all values remained lower than those of the CK. Under T2D2, NDVInb/2D-Region/ASM reached a minimum of 0.436 across all D2 treatments. By D3, the values across all the temperatures slightly exceeded those of the CK, but the differences were not significant. Fig. 6(E) reveals that qP/1D-Parallel/TENT generally increases and then decreases with prolonged cold stress, remaining higher than that of the CK during D1 and D2. At D3, the qP/1D-Parallel/TENT at T2 was 30.93 ​% greater than that at CK, whereas at T3 and T4, it decreased significantly to 72.18 ​% and 82.89 ​% of that of CK, respectively. Finally, Fig. 6(F) shows that Y (Ⅱ)/1D-Parallel/ASM slightly increased as the temperature gradient decreased under the D1 treatment. A marked reduction in Y (Ⅱ)/1D-Parallel/ASM was observed between T2D2 and T3D2, while the levels across all the temperature treatments at D3 were the lowest overall compared with those at D1 and D2.

### 3.3 Inversion of the Photosynthetic Physiological Potential Index in strawberry plants

**Fig. 7:**
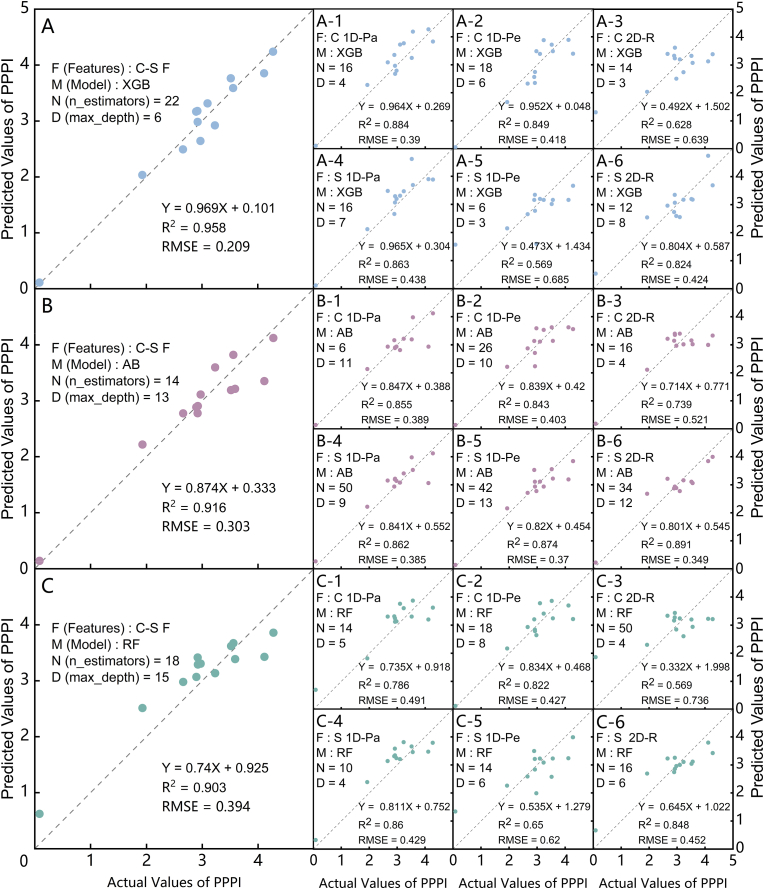
Inversion modeling schemes and accuracy validation of the PPPI based on different models and phenotypic spatial variability features: (A), (B), (C) represent the accuracy fitting of the PPPI prediction models using XGBoost (XGB), AdaBoost (AB), and Random Forest (RF), respectively, with key spatial variability features from multi-source phenotypic parameters as inputs; (A-1 to A-3), (B-1 to B-3), (C-1 to C-3) represent the accuracy fitting of PPPI prediction models using typical spatial variability features from chlorophyll fluorescence parameters in 1D-Parallel (C 1D-Pa), 1D-Perpendicular (C 1D-Pe), and 2D-Region (C 2D-R) as inputs; (A-4 to A-6), (B-4 to B-6), (C-4 to C-6) represent the accuracy fitting of PPPI prediction models using typical spatial variability features from hyperspectral index parameters in 1D-Parallel (S 1D-Pa), 1D-Perpendicular (S 1D-Pe), and 2D-Region (S 2D-R) as inputs.

Fig. 7(A), (B), and (C) illustrate the predictive performance of the XGBoost (XGB), AdaBoost (AB), and RandomForest (RF) models for the Photosynthetic Physiological Potential index (PPPI) based on chlorophyll fluorescence phenotypes, hyperspectral phenotypes, and multi-source key phenotypic fusion across different dimensions and distribution orientations of strawberry leaves, respectively. The results indicate that when the Chlf-Spec Fusion features (C-S F) were used as the inputs, the XGB model achieved the closest distribution of the predicted PPPI values to the 1:1 line, with a coefficient of determination (R^2^) of 0.958 and a root mean square error (RMSE) of 0.209. The RF-based model exhibited lower predictive accuracy than XGB and AB, with an R^2^ of 0.903 and an RMSE of 0.394, indicating greater deviation in predicting lower PPPI values.

Fig. 7(A-1∼6), (B-1∼6), and (C-1∼6) present the performance of the PPPI inversion models when spatial variation features from single-type phenotypic data across dimensions and directions were used under the different algorithms. Notably, the MI value of NDVIng/1D-Perpendicular/CON ranked among the top two for both P_max_ and Chl_a ​+ ​b_. Therefore, this feature was retained as a representative feature for Chl_a ​+ ​b_, whereas for P_max_, the third-ranked feature, NDVI/1D-Perpendicular/TENT, was used to ensure the inclusion of six key features. Similarly, for REC in the 2D-Region, the feature NDVIgb/CON was included to supplement the analysis.

The XGB models, which used the typical spatial variability features from the chlorophyll fluorescence (Chlf) imaging in the 1D-Parallel dimension and hyperspectral (Spec) imaging in the 1D-Parallel dimension yielded R^2^ values exceeding 0.85. However, the XGB models that used the Chlf 2D-Region and Spec 1D-Perpendicular spatial variability features as input parameters exhibited larger deviations in predicting both low and high PPPI values than the AB models. The AB model achieved an R^2^ of 0.891 and an RMSE of 0.349 when the Spec 2D-Region spatial variability features were used, with most prediction errors occurring in the mid-range PPPI values. Compared with the XGB or AB models, the RF models, when single-dimensional and direction-distributed spatial variability features were used as inputs, consistently underperformed. Specifically, the RF model using the spatial variability features of the Chlf 2D_Region showed significant deviations in both low and high PPPI value predictions, with an R^2^ of 0.569 and an RMSE of 0.736, the lowest accuracy and alignment with the 1:1 line among all the models. The Chlf 2D-Region spatial variability features performed poorly across all three tree-based ensemble models.

### 3.4 Prediction of the strawberry cold damage risk index fusing phenotypic spatial features

#### 3.4.1 Construction of the strawberry cold damage risk index

As shown in Table 7, the PPPI varied under different levels of cold stress and durations. Under the T2D2 treatment, the PPPI initially showed a low value but later recovered to a relatively high level as the stress continued, indicating that the PPPI under T2D2 did not effectively reveal the subsequent risk of cold damage in strawberries. Similarly, the PPPI under T4D2 increased significantly compared with that under the D1 treatment but then decreased toward zero in the D3 treatment. These findings suggest that the PPPI reflects only the current photosynthetic physiology potential of strawberry plants without capturing latent information regarding future physiological changes.

**Fig. 8:**
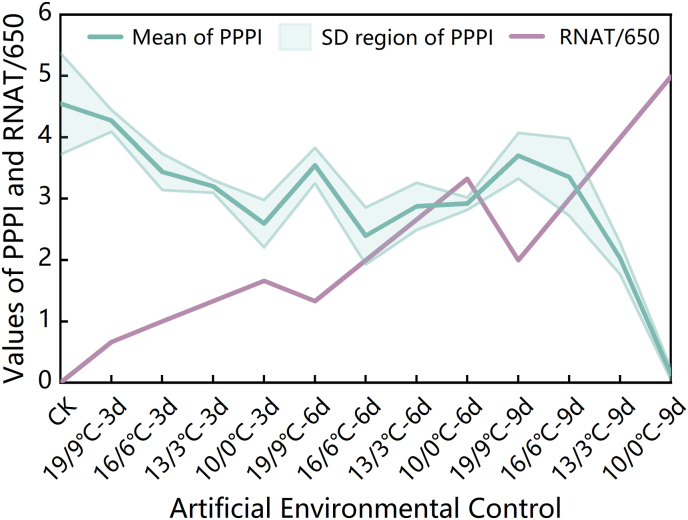
Changes in the PPPI and RNAT/650 under the different controlled environment treatments.

Relative Negative Accumulated Temperature (RNAT) is an index that monotonically decreases as the temperature decreases and the duration of exposure increases. As illustrated in Fig. 8, a comparative analysis between the PPPI curve and the RNAT/650 curve across the different controlled environment treatments revealed that the distance between these two curves can indicate the stress level experienced by the strawberry plant under cold stress. The intersection point of these curves effectively marks the critical threshold where cold damage occurred in the strawberries.

**Fig. 9:**
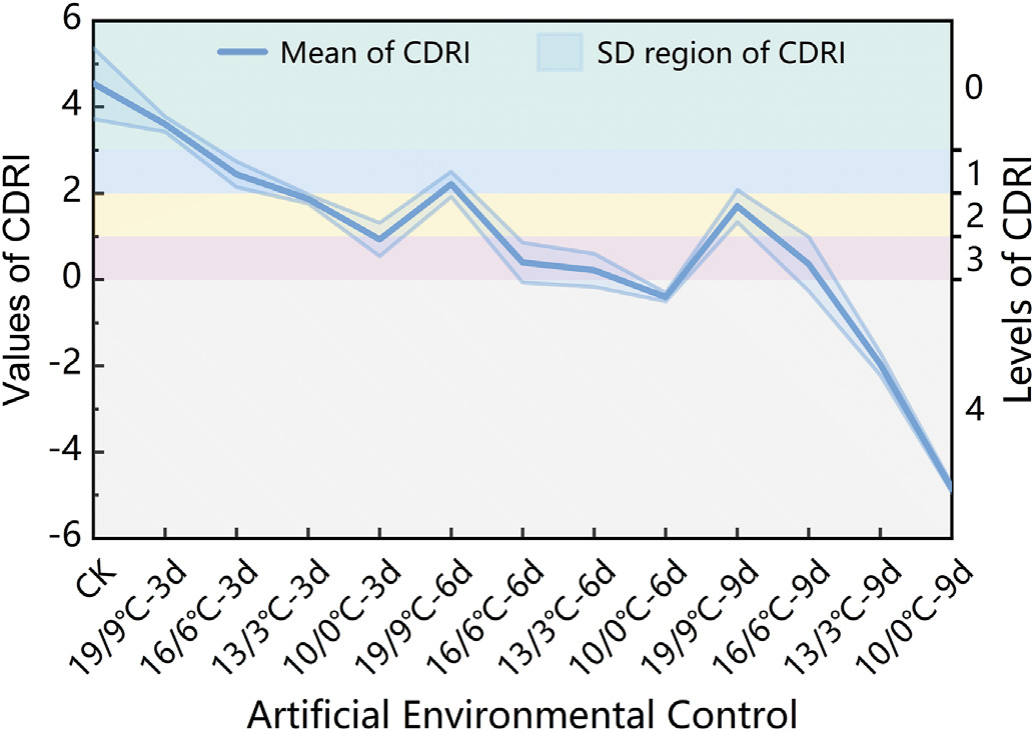
Changes in and levels of CDRI under the different controlled environment treatments.

Based on this observation, we defined the difference between PPPI and RNAT/650 as the Cold Damage Risk Index (CDRI) for strawberries, which can be expressed as follows: CDRI=PPPI−RNAT/650. The CDRI curves for the different controlled environment treatments are shown in Fig. 9. The CDRI not only reflects the overall trend of the PPPI in response to varying levels of cold stress but also shows a monotonic relationship with decreasing environmental temperature and increasing stress duration. The variation in the CDRI suggests that it is an effective indicator for monitoring and early warning of cold damage in strawberries. For its practical application in strawberry production, we categorized the CDRI into five levels: Level 0 (CDRI ≥3) indicates no risk of cold damage; Level 1 (2 ​≤ ​CDRI <3), Level 2 (1 ​≤ ​CDRI <2), and Level 3 (0 ​≤ ​CDRI <1) correspond to blue, yellow, and red warnings for cold damage, respectively; and Level 4 (CDRI <0) signifies that cold damage has already occurred. These levels are represented by the green, blue, yellow, red, and gray regions in Fig. 9, respectively.

#### 3.4.2 Inversion of the relative negative accumulated temperature in strawberry

**Fig. 10:**
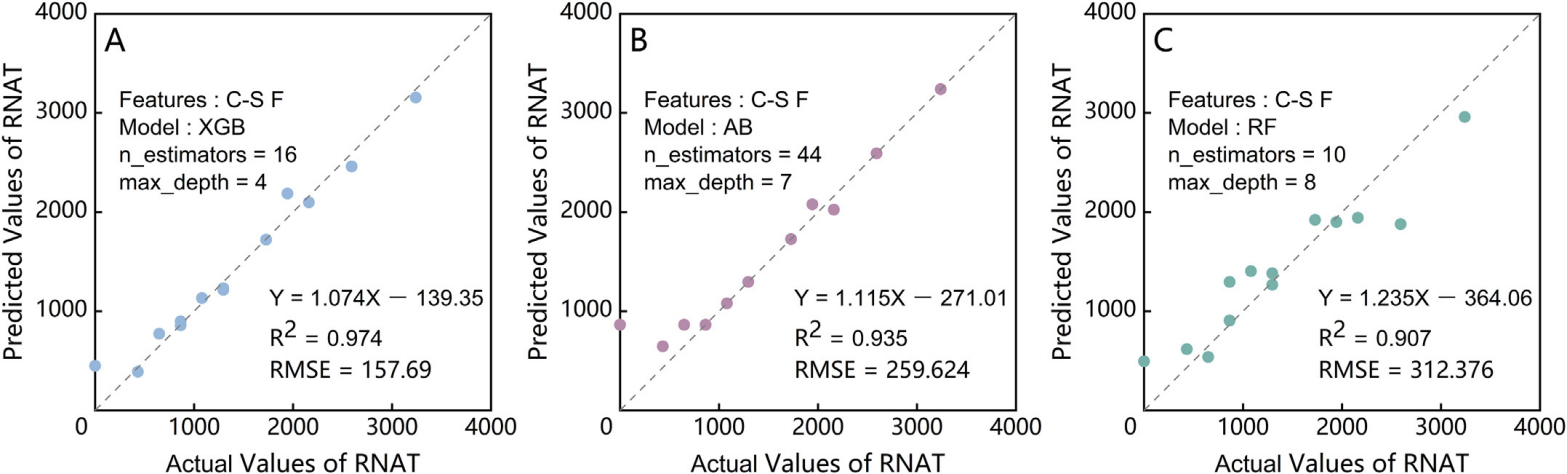
RNAT prediction accuracy and validation for the different models: (A), (B), and (C) display the performance of XGBoost (XGB), AdaBoost (AB), and Random Forest (RF), respectively, using Chlf-Spec Fusion (C-S F) multi-source phenotypic spatial variability features as the input parameters for the RNAT prediction.

To achieve a phenotype-based early warning system for cold damage risk in strawberries, it is necessary to establish a model for predicting RNAT based on phenotypic traits. In practical applications, it is important to minimize the required types and quantity of input data while maintaining accuracy. Therefore, the six key features of multi-source phenotypic spatial variability (Chlf-Spec Fusion) introduced in Section 3.2.2 were used as input parameters for RNAT prediction. As shown in Fig. 10, the performances of the three modeling algorithms ranked in terms of R^2^, RMSE, and alignment with the 1:1 line were XGB, AB, and RF, respectively, from best to worst. XGB and AB exhibited comparable performance in predicting RNAT values above 800, whereas AB showed greater deviations than XGB for values below 800. In contrast, the RF predictions were more dispersed around the 1:1 line, with the lowest R^2^ among the three models.

#### 3.4.3 Validation of the cold damage risk index and risk level accuracy

**Fig. 11:**
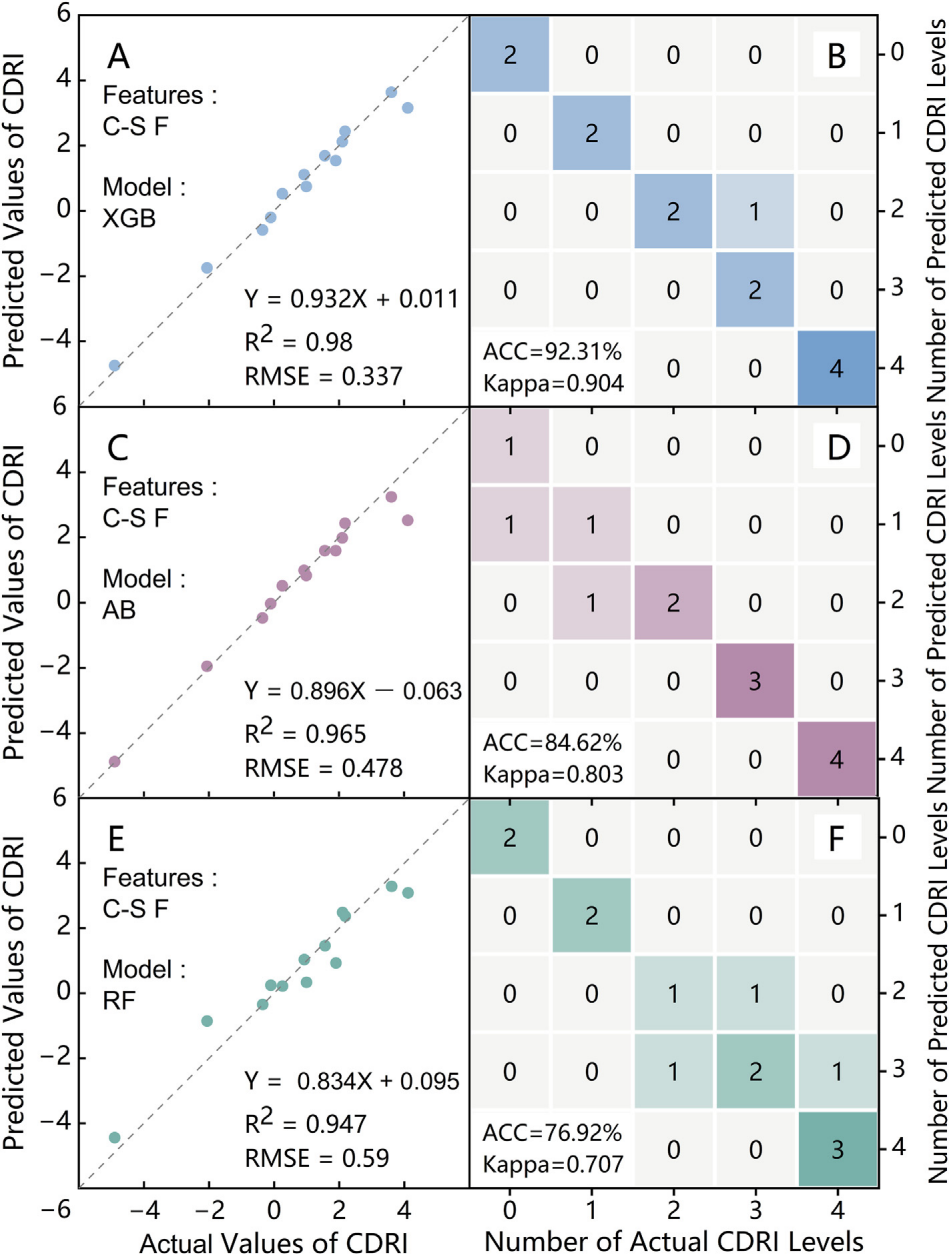
Validation of the strawberry cold damage risk index and risk level accuracy for the different models: (A) and (B), (C) and (D), (E) and (F) show the accuracy of the CDRI prediction and risk level classification using XGBoost (XGB), AdaBoost (AB), and Random Forest (RF) with Chlf-Spec Fusion (C-S F) multi-source phenotypic spatial variability features as the input parameters.

Based on the PPPI and RNAT predictions via the XGB, AB, and RF models established in Sections 3.3, 3.4.2 with multi-source phenotypic spatial variability features, the CDRI values and risk levels for the strawberry plants were calculated and validated using sample data. The accuracy evaluation of each model is presented in Fig. 11. As shown in the left column of the figure, the CDRI prediction accuracy was highest for the XGB model when the Chlf-Spec Fusion features were used as the input parameters, outperforming both the AB and RF models. The three models yielded R^2^ values greater than 0.9 for the CDRI predictions, indicating that the Chlf-Spec Fusion spatial variability features effectively captured the early potential for cold damage in the strawberries. The confusion matrix displayed on the right of Fig. 11 indicates that the accuracies (ACC) of the risk level predictions for the XGB, AB, and RF models were 92.31 ​%, 84.62 ​%, and 76.92 ​%, respectively, with Kappa coefficients of 0.904, 0.803, and 0.707. Misclassifications in the XGB and RF models were concentrated in the middle to low CDRI values, whereas AB struggled more with high CDRI values. Three integrated machine learning algorithms with multi-source phenotypic spatial features effectively predicted the CDRI, confirming the feasibility of quantifying strawberry cold stress risk using a purely phenotypic approach.

### 3.5 Interpretation of models for predicting PPPI and RNAT

**Fig. 12:**
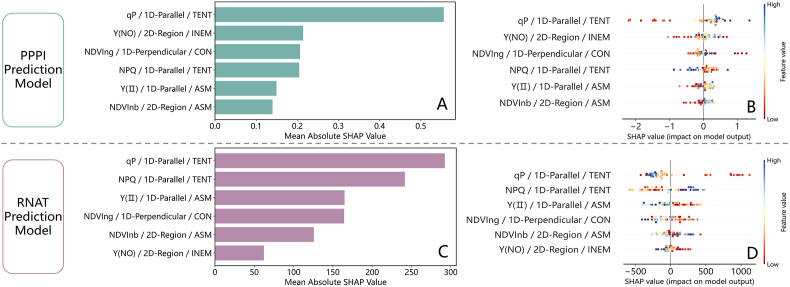
Analysis of feature contributions in strawberry PPPI and RNAT phenotypic prediction models based on XGB: (A) and (B), (C) and (D) show the absolute mean SHAP values and beeswarm plots of the key multi-source phenotypic spatial variability features in the PPPI and RNAT prediction models, respectively.

Fig. 12 displays the SHAP summary plot, which ranks the key phenotypic spatial variability features based on their importance in influencing model outputs. qP/1D-Parallel/TENT were the most important features in both the PPPI and RNAT prediction models, with average absolute SHAP values exceeding 0.5 and nearly 300, respectively. Lower values of qP/1D-Parallel/TENT corresponded to lower SHAP values in the PPPI model and higher SHAP values in the RNAT model, reinforcing the opposite trends observed in PPPI and RNAT. The average absolute SHAP values of Y(NO)/2D-Region/INEM, NDVIng/1D-Perpendicular/CON, and NPQ/1D-Parallel/TENT in the PPPI prediction model were similar, approximately 0.2, with contributions aligning with the sign of the feature values. However, their dependence was significantly lower than that of qP/1D-Parallel/TENT. NPQ/1D-Parallel/TENT exhibited a more distinct linear trend in its impact on PPPI predictions, primarily showing a negative correlation. The RNAT prediction model ranked second only to qP/1D-Parallel/TENT, with lower values contributing most negatively to the RNAT predictions, whereas higher values exerted a positive influence, albeit to a lesser extent than qP/1D-Parallel/TENT. Y(NO)/2D-Region/INEM had the least impact on the RNAT predictions, with an average absolute SHAP value of less than 20 ​% of the highest contributing feature.

**Fig. 13:**
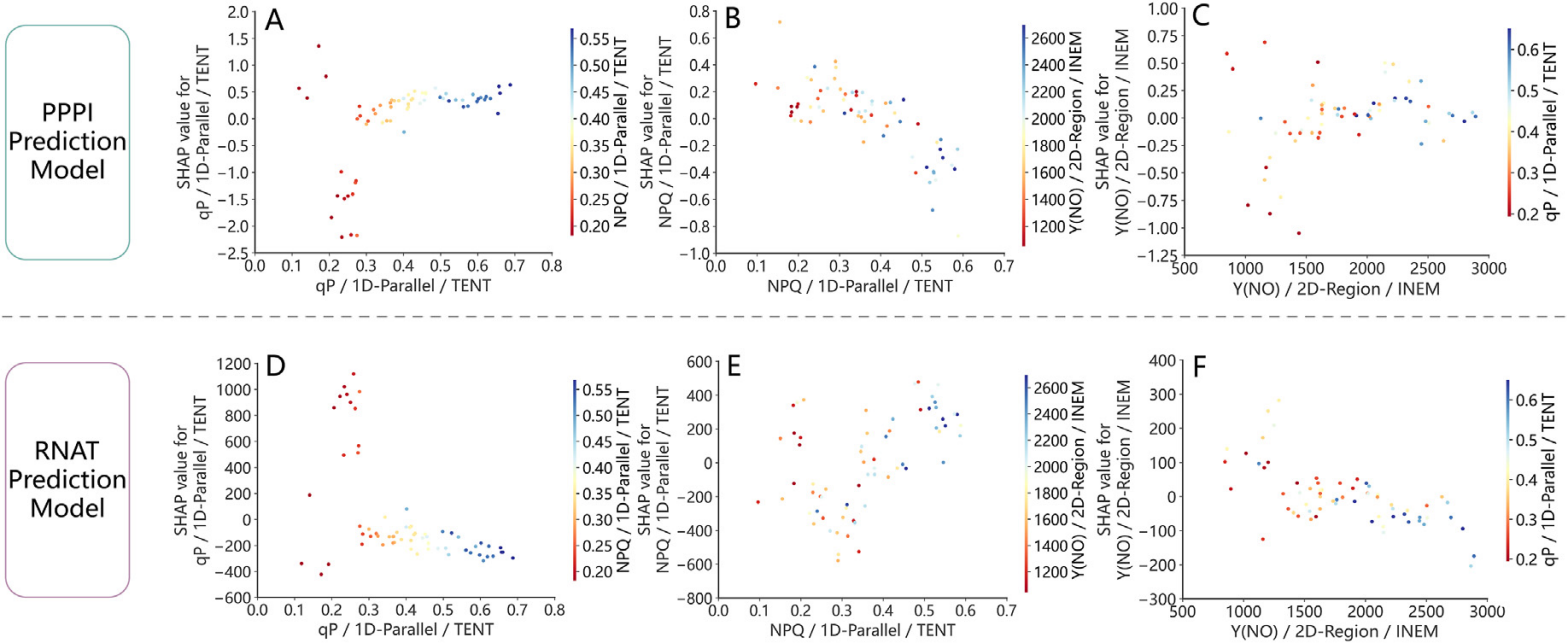
Analysis of the patterns of influence and dependencies of the selected features in the phenotypic prediction models for PPPI and RNAT: (A) and (D) depict the dependency of the texture entropy of qP along a line parallel to the main leaf vein on the texture entropy of NPQ along a line parallel to the main leaf vein and their impact on the PPPI and RNAT prediction models; (B) and (E) depict the dependency of the texture entropy of NPQ along a line parallel to the main leaf vein on the inertia moment of Y(NO) in the 2D leaf region and their impact on the PPPI and RNAT prediction models; (C) and (F) depict the dependency of the inertia moment of Y(NO) in the 2D leaf region on the texture entropy of qP along a line parallel to the main leaf vein and their impact on the PPPI and RNAT prediction models.

Fig. 13 further illustrates the patterns of influence and dependencies of the selected phenotypic spatial variability features within the XGB-based models for predicting PPPI and RNAT. A threshold of approximately 0.28 in qP/1D-Parallel/TENT marked a turning point in its contribution to the PPPI and RNAT prediction models. Below this threshold, qP/1D-Parallel/TENT had a strong negative effect on PPPI and a significant positive effect on RNAT. When the feature value exceeded 0.28, its positive influence on PPPI and negative influence on RNAT progressively intensified. The critical threshold for NPQ/1D-Parallel/TENT in the PPPI prediction model was approximately 0.4, beyond which its negative impact intensified at an increasing rate. In the RNAT prediction model, NPQ/1D-Parallel/TENT shifted from a positive influence to a negative influence as its value increased, with the inflection point occurring between 1400 and 2000. Additionally, qP/1D-Parallel/TENT and NPQ/1D-Parallel/TENT exhibited strong positive dependencies with clear linear trends. While Y(NO)/2D-Region/INEM also correlated positively with qP/1D-Parallel/TENT, its dependency was weaker than that between qP/1D-Parallel/TENT and NPQ/1D-Parallel/TENT.

## 4 Discussion

Cold stress is a significant challenge for strawberry cultivation throughout its entire growth cycle. Compared with those of the seedling and maturation stages, the flowering and fruiting stages of strawberries have longer physiological development periods. In practical production, strawberry plants typically continue flowering and fruiting throughout the entire growing season, underscoring the critical need for effective cold stress management during this period [61]. With the rapid advancements in sensor and computing technologies, considerable progress has been made in plant physiological data collection and analysis methods, driving agricultural meteorological risk management toward greater precision, convenience, and comprehensiveness. While plant imaging-based phenotypic physiological assessments have been widely reported, to date, no studies have quantitatively analyzed the contribution of directional heterogeneity in plant imaging phenotypes to physiological evaluations or explored the fusion of multi-source spatial variability features for disaster monitoring and early warning. Therefore, this study focused on the flowering and fruiting stages of strawberry plants, with the aim of fully utilizing multi-source phenotypic spatial information to identify characteristic parameters that reflect changes in photosynthetic physiological activity under cold stress. By fusing key phenotypic features through machine learning algorithms, this research aims to develop a purely phenotype-based cold stress monitoring and risk warning system for strawberries, providing new theoretical support and scientific strategies for cold stress prevention and damage assessment in modern strawberry agriculture.

Initially, this study examined the changes in photosynthetic physiological activity under different cold stress gradients and durations by analyzing P_max_, REC, and Chl_a ​+ ​b_. Under all the cold stress gradients, the trend of P_max_ initially decreased and then increased with prolonged stress exposure, which is consistent with observations by Öquist [62]. This phenomenon may be attributed to the activation of protective mechanisms within the plant's photosynthetic system during the early stages of cold stress, which limits electron transport rates and stomatal opening [63]. The higher P_max_ observed under the T3D2 and T4D2 conditions than under the T1D2 and T2D2 conditions might indicate that the plants triggered stronger physiological adaptations, such as increased Rubisco enzyme activity, antioxidant production, and hormone regulation, resulting in a paradoxical increase in P_max_ [64]. Chl_a ​+ ​b_ levels decreased with prolonged stress duration under the T1 to T3 cold gradients, whereas significant increases were observed under T4D1 and T4D2. The factors leading to elevated chlorophyll levels under cold stress may include dehydration-induced increases in chlorophyll density per unit leaf mass or the activation of plant repair mechanisms to maintain photosynthetic capacity. Water loss can cause the phospholipid bilayer of the cell membrane to become more compact, reducing fluidity and increasing electrolyte leakage [65]. Notably, the REC under T4D1 did not significantly differ from that under T3D1 and T2D1, suggesting that the integrity of the cellular membrane structure was similar, with strawberry plants under T4D1 compensating for reduced photosynthetic rates by increasing chlorophyll synthesis or reducing chlorophyll degradation.

PCA is a widely recognized scientific method for dimensionality reduction and has found extensive applications in various fields, such as image processing and clinical research [66]. In this study, we employed PCA to reduce the dimensionality of the information contained within the three photosynthetic physiological parameters—P_max_, REC, and Chl_a ​+ ​b_—compressing them into a single metric, the PPPI, which fuses data on the maximum photosynthetic rate, plasma membrane structure, and chlorophyll content. Analysis of the average levels of and changes in the PPPI under the different cold stress gradients and durations revealed that the PPPI demonstrated greater robustness in reflecting cold stress compared to any single photosynthetic parameter. It also effectively reflected the regulatory and compensatory changes in strawberry photosynthetic physiology under the T2D2 and T4D2 conditions. However, this also highlights a limitation of PPPI—its non-monotonicity under escalating cold stress. Specifically, the PPPI reflects the photosynthetic potential of a plant at a given moment without predicting the physiological inertia of the strawberry plants. In contrast, RNAT incorporates both temporal and thermal dimensions, showing monotonicity and inertia in its changes. While RNAT is a necessary but insufficient condition for cold damage occurrence, balancing PPPI and RNAT allowed us to construct the CDRI. The CDRI captured the typical trends of strawberry photosynthetic potential under varying degrees of cold stress while also maintaining monotonic inertia across temperature and time dimensions. This index effectively characterized the cumulative risk and inertia of cold damage in strawberries.

We further applied MI analysis—a popular method in medical feature analysis—to investigate the interactions between P_max_, REC, Chl_a ​+ ​b_, and 624 features derived from 16 spatial variability characteristics across 13 imaging phenotypic parameters in 3 distributional dimensions [67]. The MI analysis revealed that spatial variability features based on chlorophyll fluorescence imaging generally presented higher MI values with photosynthetic physiological parameters than those based on hyperspectral index imaging. This result was expected, as the dynamics of chlorophyll fluorescence induction kinetics serve as an instantaneous probe of PSII functional status, reflecting real-time energy, and electron flow [68]. Conversely, variations in reflectance spectra stem from changes in chlorophyll content, leaf water content, thylakoid membrane stacking, and leaf surface properties—processes that require time to manifest [69]. This study employed the normalized difference vegetation index (NDVI) formula, which combines four representative spectral bands in a pairwise fashion, to characterize changes in strawberry leaf spectral phenotypes under cold stress. This approach not only eliminates absolute reflectance variations caused by external uncertainties such as leaf inclination and uneven illumination but also enhances signal differentiation between target bands [70]. We observed that the MI values between the NDVI in the 1D-Perpendicular dimension and P_max_, REC, and Chl_a ​+ ​b_ were low for metrics such as A, VAR, and COV. However, the MI values for NDVIgb and NDVIgr on the same 1D-Perpendicular dimension with photosynthetic parameters were notably greater. We speculate that the instability in NIR reflectance under cold stress in the 1D-Perpendicular direction is due to the influence of factors such as thylakoid membrane stacking or leaf water content. Previous studies have highlighted the crucial role of the main vein in photosynthetic efficiency and water conduction, functions that may be significantly impacted by stress conditions such as cold, potentially reflected in directional distribution [71]. Another intriguing observation was the near-zero MI between Y(NO)/2D-Region/A and the photosynthetic parameters, whereas the mean MI in the 1D-Parallel and 1D-Perpendicular directions exhibited more pronounced interactions with physiological changes. Furthermore, the MI values for Y(NO) in the 2D-Region dimension with metrics such as INE and INEM were significantly greater than those in the 1D-Parallel and 1D-Perpendicular dimensions. This suggests that the inertia of Y(NO) within the 2D-Region is related to P_max_, REC, and Chl_a ​+ ​b_, whereas the low MI between Y(NO)/2D-Region/A and the photosynthetic parameters indicates a strong overall PSII regulatory capacity of the leaf. In contrast, the Y(NO) characteristics in the 1D-Parallel and 1D-Perpendicular directions reflect localized, unstable, high-frequency traits. Moreover, the MI value between the Chlf 2D-Region and P_max_ was lower than that of Spec 1D-Perpendicular. We attribute this to the lower water content in regions farther from the primary vein, while the 1D-Perpendicular spatial feature spans both sides of the leaf, allowing normalized spectral indices incorporating near-infrared bands to capture the water distribution along the perpendicular direction to the primary vein. Additionally, neither the fluorescence parameters nor the spectral indices presented the highest MI with P_max_ within the 2D-Region spatial domain, suggesting that the 2D-Region weakens spatial variability features across different data sources, dimensions, and orientations.

The six key phenotypic spatial variability features extracted from the strawberry plants were identified as texture features, including two TENT features, two ASM features, and one each for CON and INEM. These features were distributed across three different spatial orientations: three in the 1D-Parallel direction, one in the 1D-Perpendicular direction, and two in the 2D-Region. The primary phenotypic image data were derived from four chlorophyll fluorescence induction kinetics parameters and two hyperspectral indices, indicating that the texture features of chlorophyll fluorescence parameters in the 1D-Parallel direction are closely related to photosynthetic physiological changes under cold stress. TENT reflects the randomness in the gray-level distribution of an image, where higher entropy indicates a more complex gray-level distribution and lower entropy signifies a smoother distribution [72]. The TENT values for qP and NPQ in the 1D-Parallel direction initially increased but then decreased under the various cold stress treatments. We attribute this to the early changes in the efficiency of the photosynthetic electron transport chain and the pigment content in certain regions of the leaf, leading to increased heterogeneity. With prolonged stress, the quenching characteristics in the remaining leaf regions adjusted according to the stress level, causing the spatial distributions of qP and NPQ to return to a more uniform state. ASM measures the consistency of texture in grayscale images, with higher values indicating a more uniform gray-level distribution [73]. The 2D-Region distribution of NDVInb also exhibited a transition from disorder to order as the duration of cold stress increased. In combination with the observed trends in P_max_, REC, and Chl_a ​+ ​b_, we infer that mild cold stress has a minimal effect on the physiological processes of the plant, maintaining high complexity in the internal leaf tissue structure. In contrast, more severe cold stress tends to homogenize plant tissues, resulting in greater spectral feature consistency. As cold stress intensified, Y (Ⅱ) displayed increased heterogeneity in the 1D-Parallel direction, likely due to the localized reduction in PSII photochemical efficiency, which led to greater fluctuations in Y (Ⅱ) across the image. Notably, the CON values for NDVIng in the 1D-Perpendicular direction were lower under the T1D3, T2D3, T3D2, and T4D2 treatments but significantly increased under the T3D3 and T4D3 treatments. This could be due to the initial phase of stress manifesting primarily as a reduction in the NIR reflectance, thereby decreasing the amplitude of the reflectance spectral fluctuations in the 1D-Perpendicular direction and reducing the gray-level differences in the NDVIng images, resulting in lower CON values. As the cold stress intensified, damage to internal leaf structures became more severe, and chlorophyll degradation reached its peak in the treatment groups. The differential degradation of chlorophyll on either side of the main vein in the 1D-Perpendicular direction might have led to increased gray-level differences in NDVIng, causing an increase in CON.

The fusion of multi-source phenotypic spatial variability features into the modeling of strawberry cold damage and risk forecasting revealed that the XGB-based model outperformed the AB and RF algorithms in predicting PPPI and RNAT, as well as in calculating the CDRI and its corresponding risk levels. Notably, the AB algorithm showed greater accuracy in predicting lower PPPI values when typical spatial variability features derived from a single phenotypic distribution dimension and direction were used. However, XGB and RF differed in their accuracy under the varying input features. When the spatial variability features of chlorophyll fluorescence and hyperspectral imaging phenotypes were fused as modeling inputs, the XGB model demonstrated superior predictive accuracy in the higher PPPI range and lower RNAT range compared to AB. This suggests that despite AB's ability to combine multiple weak classifiers, its fitting and generalization capabilities fall short of those of XGB when dealing with complex data features, particularly in scenarios where chlorophyll fluorescence and hyperspectral imaging phenotypes are fused [74]. The RF model, which is based on the Bagging method, lagged behind both XGB and AB in predicting PPPI, RNAT, and CDRI, highlighting that Boosting methods are better suited for handling nonlinear relationships among multiple feature combinations, a finding that is consistent with previous research [75]. Furthermore, the XGB model developed by integrating multi-source phenotypic spatial variability features outperformed Lu et al.'s [76] chlorophyll fluorescence-based model for predicting three cold injury levels in cucumbers, achieving higher prediction accuracy and a significantly higher R^2^ value than the frost damage prediction in loblolly pine seedlings using hyperspectral imaging [77]. These findings demonstrate the reliability and effectiveness of the proposed multi-source phenotypic information fusion approach for plant cold stress risk assessment.

The SHAP analysis of the XGB-based model revealed that qP/1D-Parallel/TENT was the most influential phenotypic spatial variability feature for both the PPPI and RNAT predictions, with NPQ/1D-Parallel/TENT being the second most significant feature in both models. These findings suggest that the chlorophyll fluorescence quenching coefficient and texture entropy in the main vein direction play crucial roles in indicating photosynthetic physiological changes in strawberry plants under cold stress. Further decomposition of these key features revealed that qP reflects the proportion of open reaction centers in PSII, whereas NPQ reflects a plant's ability to dissipate excess light energy through thermal dissipation. The variations in these fluorescence parameters are influenced by multiple physiological factors, including chlorophyll content, water content, the xanthophyll cycle, and reactive oxygen species. Their one-dimensional distribution along the main vein serves as a simplified expression of leaf photoprotection across the leaf from base to tip, whereas TENT, by describing the uniformity of information distribution, further reveals the dynamic responses of phenotypic physiological traits under stress. Overall, we conclude that the strawberry cold stress monitoring and early warning model constructed using phenotypic spatial variability features likely captures and quantifies the dynamic evolution of physiological traits, including chlorophyll content, water content, and the xanthophyll cycle, along the main vein under cold stress. This comprehensive approach to studying a leaf's internal physiological state—such as chlorophyll content, water content, reactive oxygen species, and stomatal conductance—along with external phenotypic traits, offers a more precise phenotypic and physiological service for advancing precision agriculture.

The conclusions of this study are based on the short-day strawberry cultivar ‘Toyonoka’. Owing to the differences in photoperiod response characteristics and cold tolerance between short-day and day-neutral/long-day strawberry varieties [78], the phenotypic traits reflecting stress response mechanisms may exhibit distinct patterns. Variations in day-night temperature differences and the occurrence of intermittent stress events could also influence the photosynthetic system's response across different growth stages, potentially altering the applicability of crop meteorological disaster monitoring and early warning systems. To further enhance the generalizability of the findings of this study, future research should involve systematic experiments across different photoperiod types of strawberry cultivars, incorporating additional environmental variables such as humidity, light intensity, photoperiod, and intermittent stress frequency. This will help explore the interactions of these factors on plant phenotypic traits. Through multivariable interaction experiments and model optimization, the knowledge base for crop meteorological disaster phenotyping and early warning can be continuously refined, providing scientific support for risk management under diverse crop varieties and environmental conditions.

## 5 Conclusion

As temperature decreases and stress duration increases, ‘Toyonoka’ strawberry shows a declining trend in P_max_ and Chl_a ​+ ​b_, while REC continues to rise. The PPPI, integrating P_max_, REC, and Chl_a ​+ ​b_, effectively reflects photosynthetic physiological potential under varying cold stress conditions but has limited ability to distinguish actual plant health from stress-induced illusions. The CDRI accurately captures photosynthetic tolerance and cumulative stress, serving as the core indicator for cold damage risk warning. Six key phenotypic spatial variability features—NPQ/1D-Parallel/TENT, NDVIng/1D-Perpendicular/CON, Y(NO)/2D-Region/INEM, NDVInb/2D-Region/ASM, qP/1D-Parallel/TENT, and Y (Ⅱ)/1D-Parallel/ASM—exhibit the strongest dependence on P_max_, REC, and Chl_a ​+ ​b_. The XGB-based model integrating these features predicts CDRI with an R^2^ of 0.98, achieving 92.31 ​% ACC and a Kappa of 0.904, outperforming AB and RF models. qP/1D-Parallel/TENT makes the strongest positive contribution in the PPPI inversion model and the strongest negative contribution in the RNAT model, making it the most critical phenotypic spatial variability feature for strawberry cold damage risk prediction. This study provides a novel phenotypic approach to assessing cold stress risk by capturing asynchronous changes in leaf-scale traits.

## Author contributions

Conceptualization, N.J. and Z.Y.; methodology, N.J. and Z.Y.; software, N.J.; validation, H.Z. and C.Z.; formal analysis, N.J.; investigation, N.J., H.Z., C.Z., C.W., and N.W.; resources, N.J., Z.Y., and C. X; data curation, N.J., N.W., and C.X.; writing—original draft preparation, N.J. and Z.Y.; writing—review and editing, H.Z., C.Z., and C.W.; visualization, N.J.; supervision, Z.Y.; project administration, N.J. and Z.Y.; funding acquisition, Z.Y., N.J., and C.X. All authors have read and agreed to the published version of the manuscript.

## Data

The original contributions presented in the study are included in the article. Further inquiries can be directed to the corresponding author.

## Funding

This work was supported by the 10.13039/501100001809National Natural Science Foundation of China [grant number 42275200]; the 10.13039/100016724Postgraduate Research & Practice Innovation Program of Jiangsu Province [grant number KYCX24_1446]; and the 10.13039/501100001809National Natural Science Foundation of China [grant number 32360443].

## Declaration of competing interest

The authors declare that they have no known competing financial interests or personal relationships that could have appeared to influence the work reported in this paper.
